# Supplementary material for: Elastomeric polyamide biomaterials with stereochemically tuneable mechanical properties and shape memory
Source: Nat Commun. 2020 Jun 26;11:3250. doi: 10.1038/s41467-020-16945-8 (PMC7320000; doi:10.1038/s41467-020-16945-8)
Supplement: Supplementary file 1 — Supplementary Information [file 41467_2020_16945_MOESM1_ESM.pdf]

Supplementary Information

Elastomeric Polyamide Biomaterials with Stereochemically Tuneable Mechanical Properties and Shape Memory

Worch et. al.

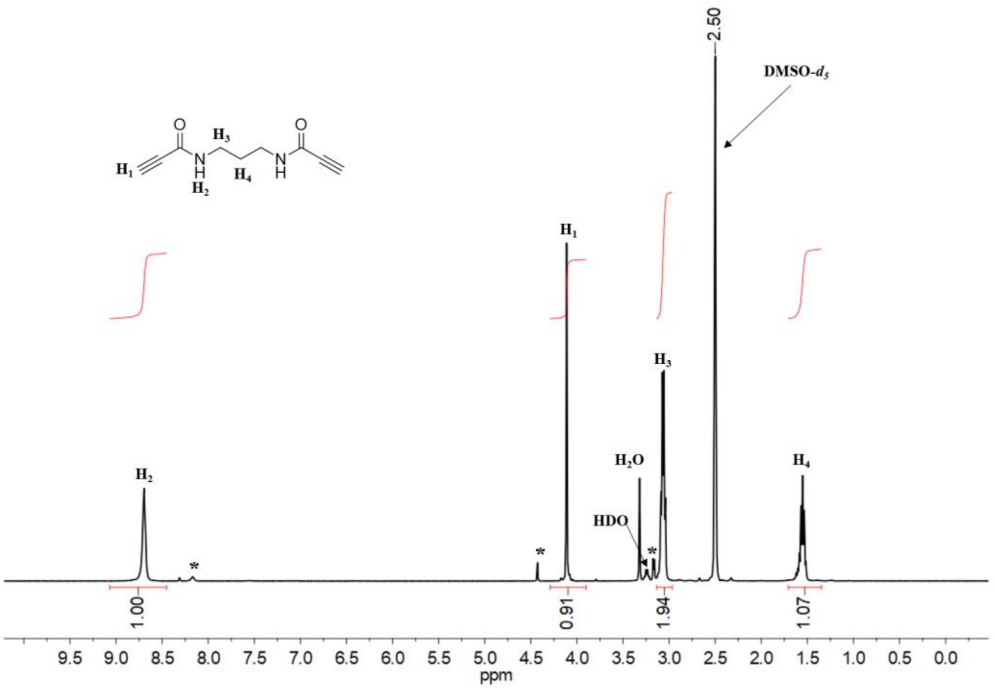

Supplementary Figure 1. Dipropiolamide (C_3A) ^{1}H NMR Spectrum – 400 MHz, DMSO-d_6. * = signals from rotamer

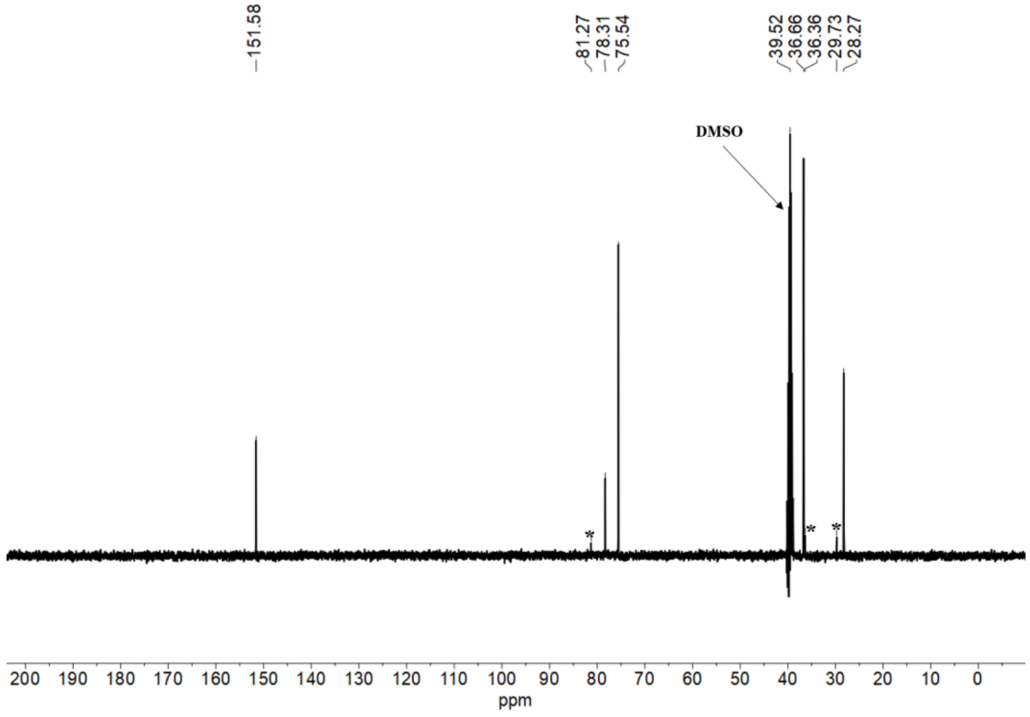

Supplementary Figure 2. Dipropiolamide (C_3A) ^{13}C NMR Spectrum – 101 MHz, DMSO-d_6. * = signals from rotamer

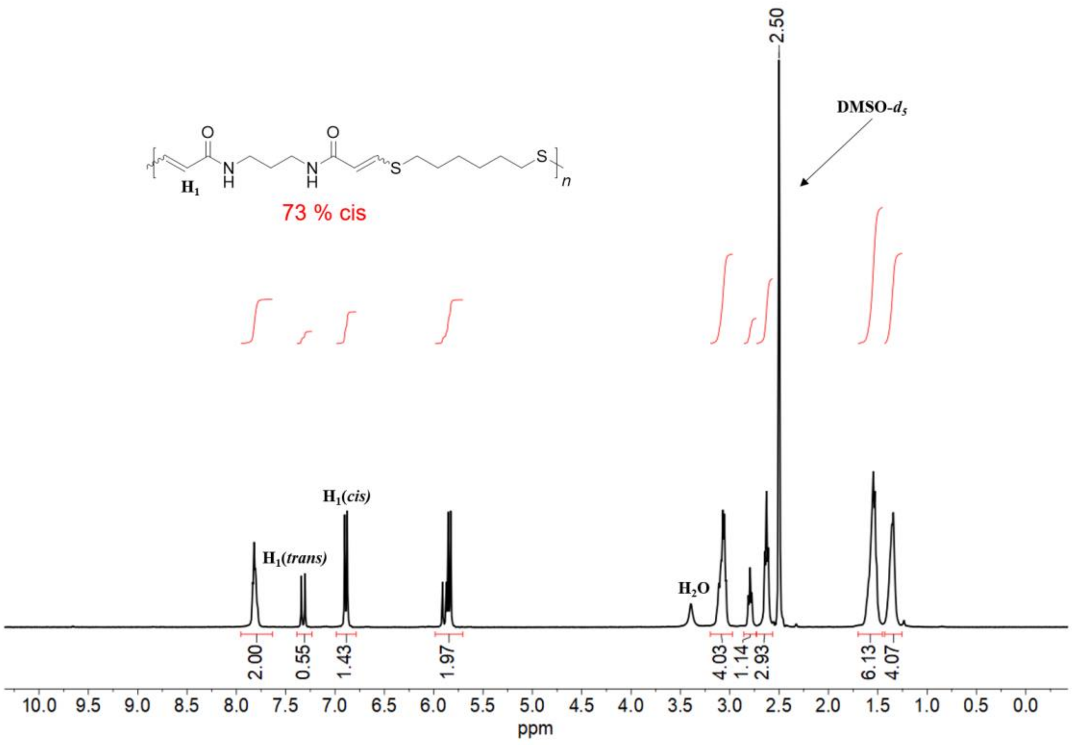

Supplementary Figure 3. C_3A-C_6T 73% *cis* ^1H NMR Spectrum – 400 MHz, DMSO-d_6.

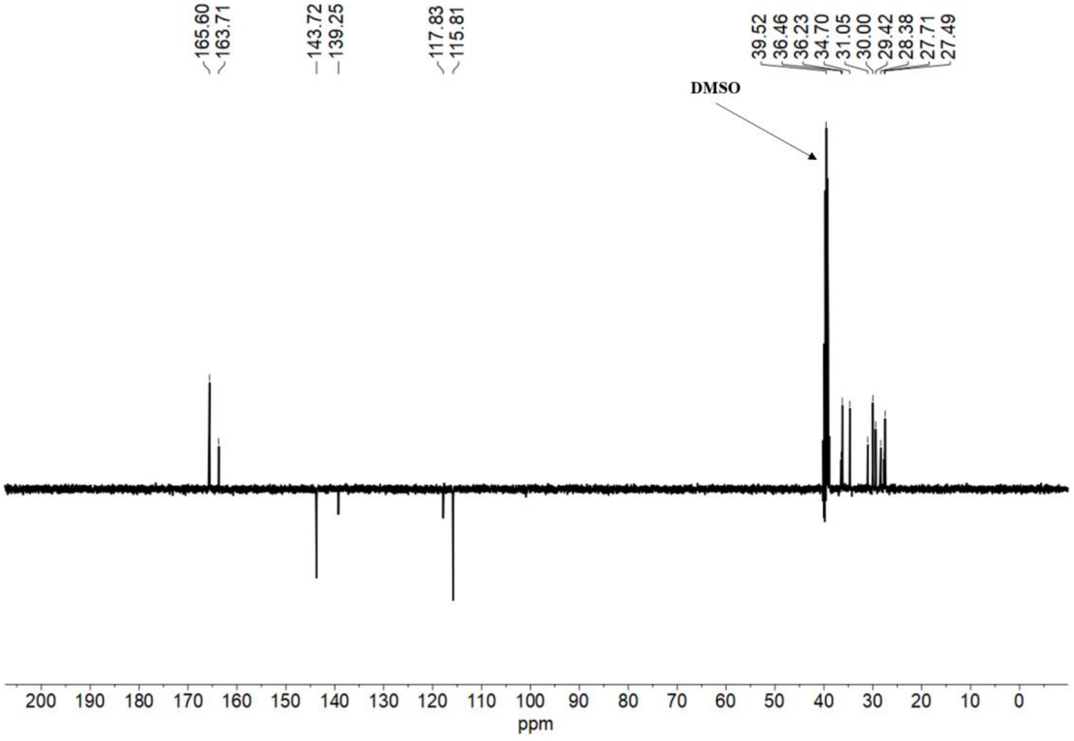

Supplementary Figure 4. C_3A-C_6T 73% *cis* ^{13}C NMR Spectrum – 101 MHz, DMSO-d_6.

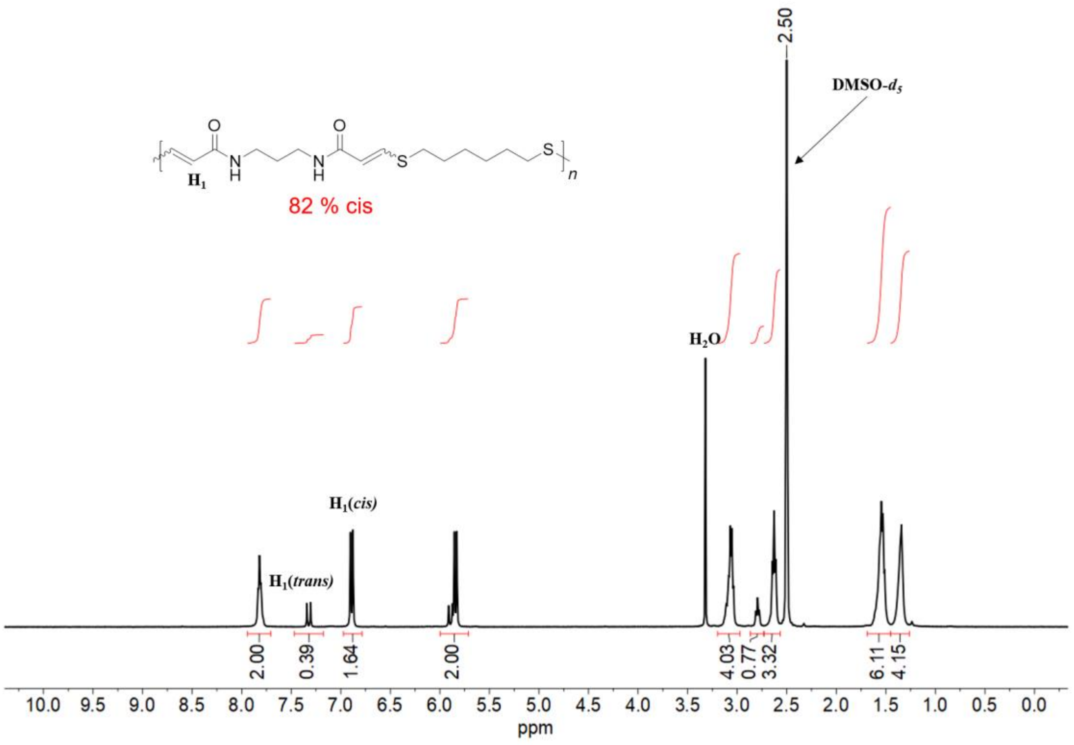

Supplementary Figure 5. C_3A-C_6T 82% *cis* ^1H NMR Spectrum – 400 MHz, DMSO-d_6.

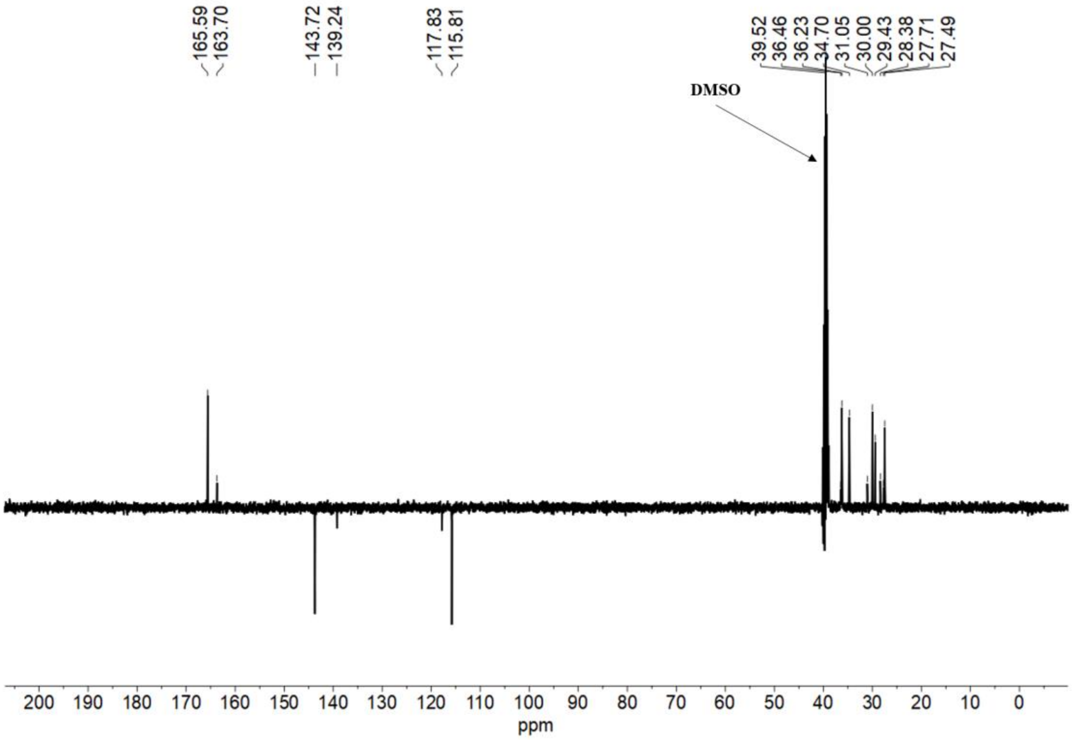

Supplementary Figure 6. C_3A-C_6T 82% *cis* ^{13}C NMR Spectrum – 101 MHz, DMSO-d_6.

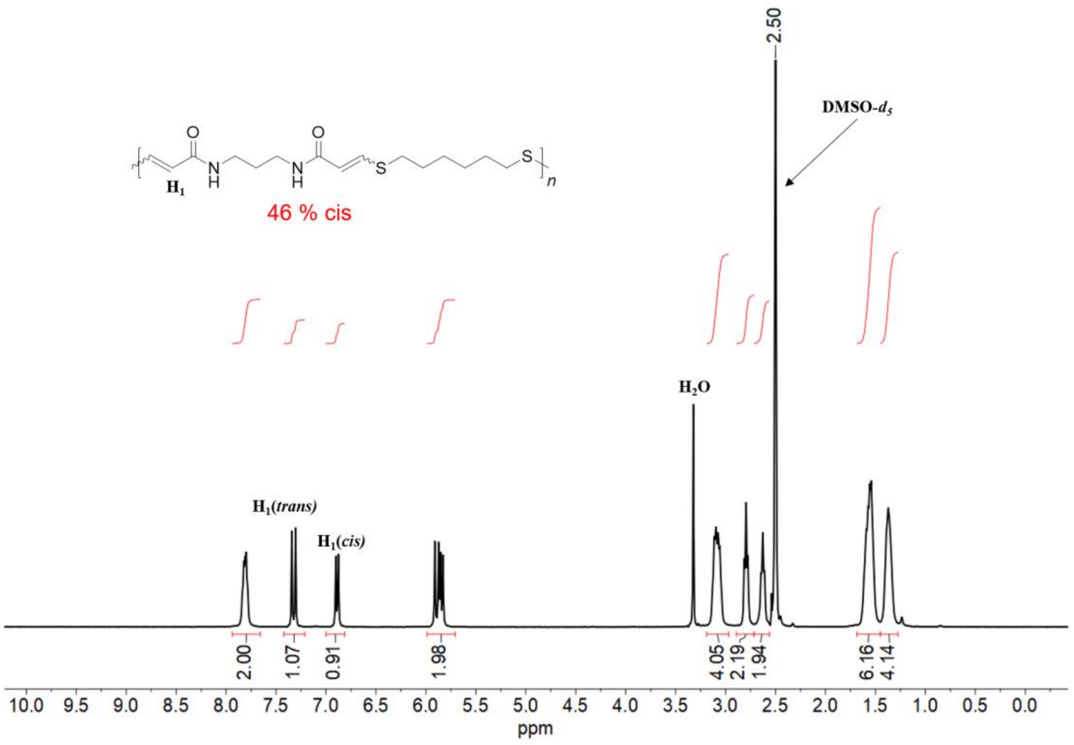

Supplementary Figure 7. C$_3$A-C$_6$T 46% *cis* ^{1}H NMR Spectrum – 400 MHz, DMSO-d_6.

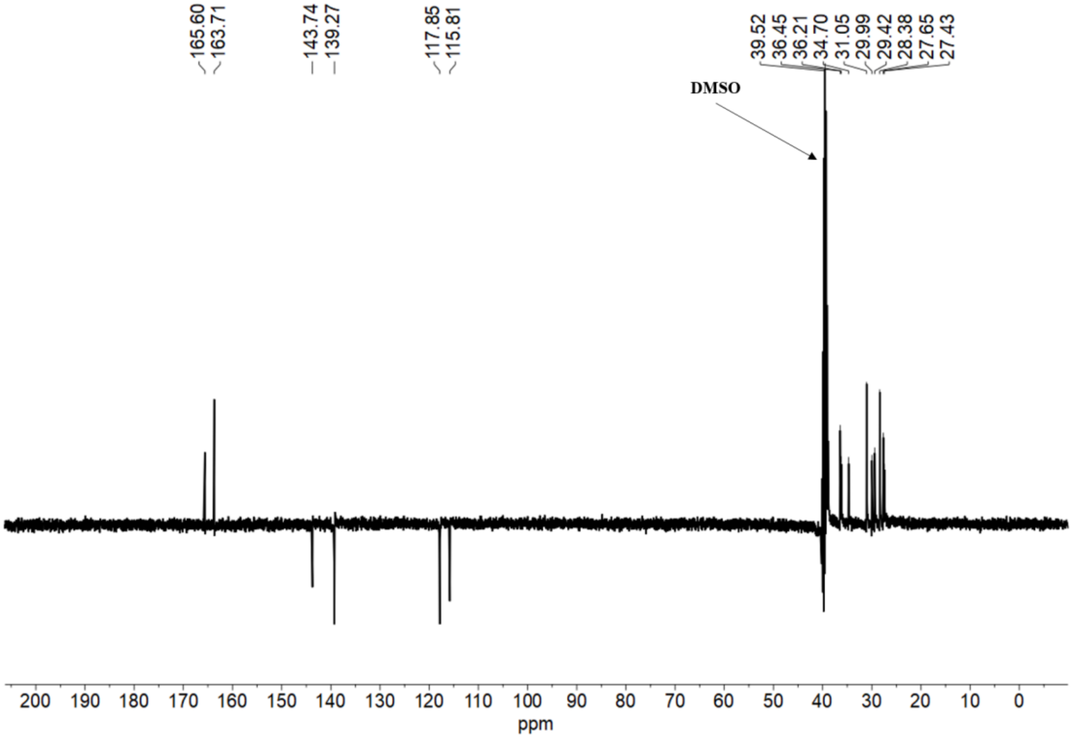

Supplementary Figure 8. C$_3$A-C$_6$T 46% *cis* ^{13}C NMR Spectrum – 101 MHz, DMSO-d_6.

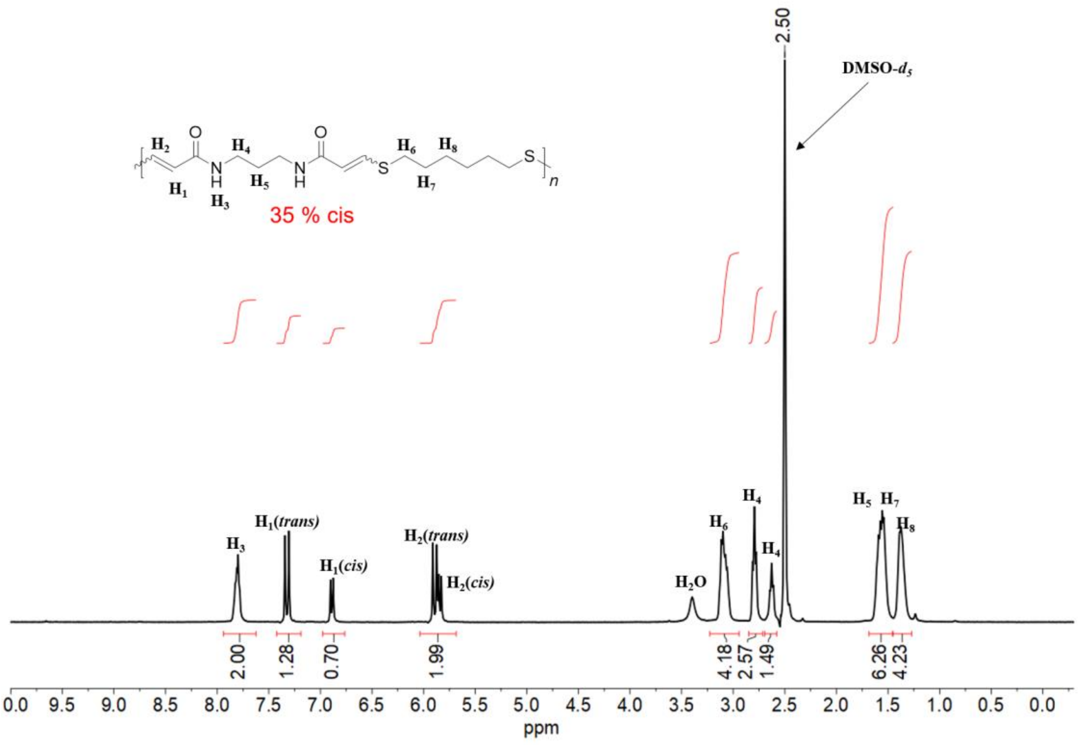

Supplementary Figure 9. C_3A-C_6T 35% *cis* ^1H NMR Spectrum – 400 MHz, DMSO-d_6.

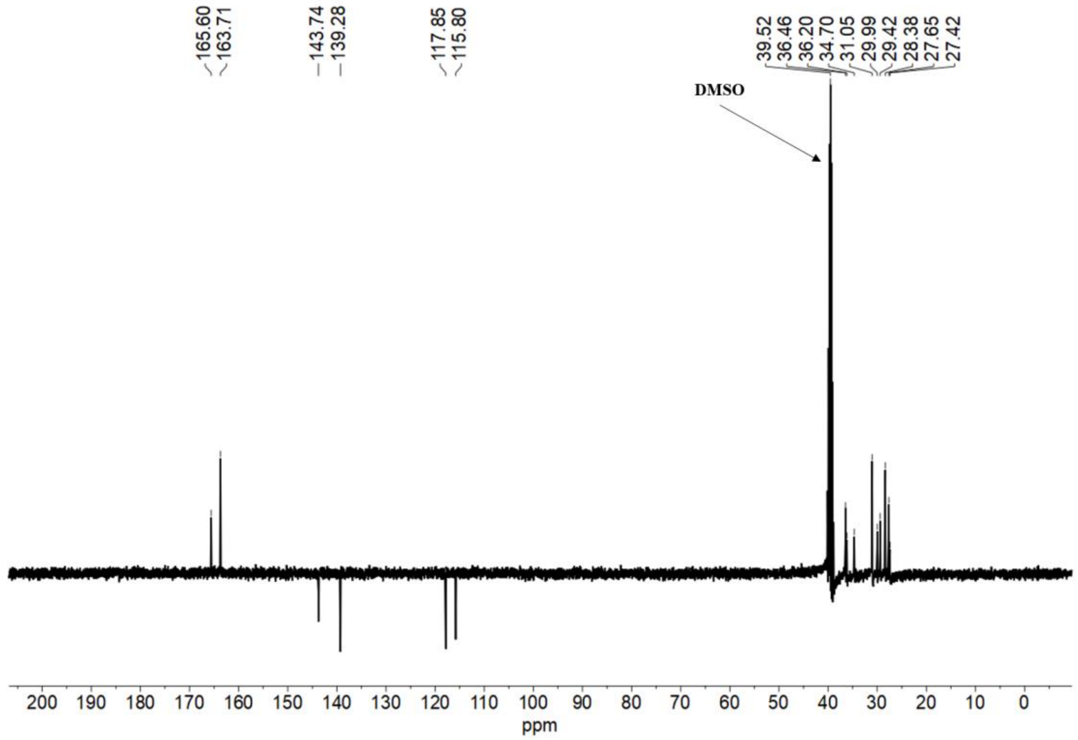

Supplementary Figure 10. C_3A-C_6T 35% *cis* ^{13}C NMR Spectrum – 101 MHz, DMSO-d_6.

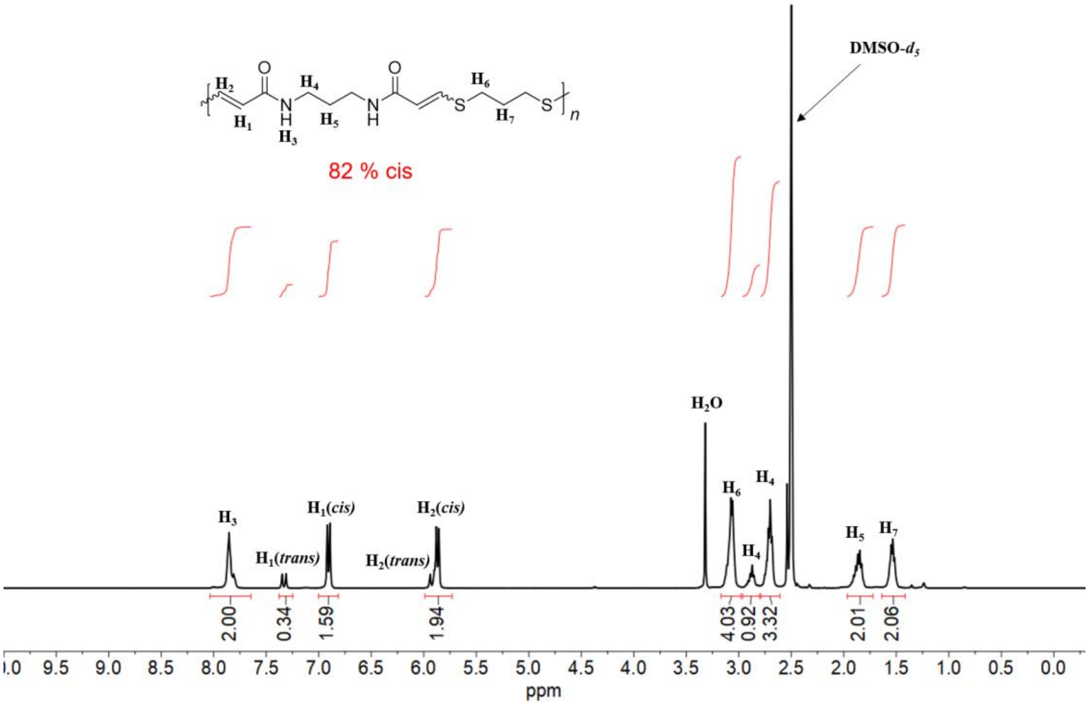

Supplementary Figure 11. C_3A-C_3T 82% *cis* ^1H NMR Spectrum – 400 MHz, DMSO-d_6.

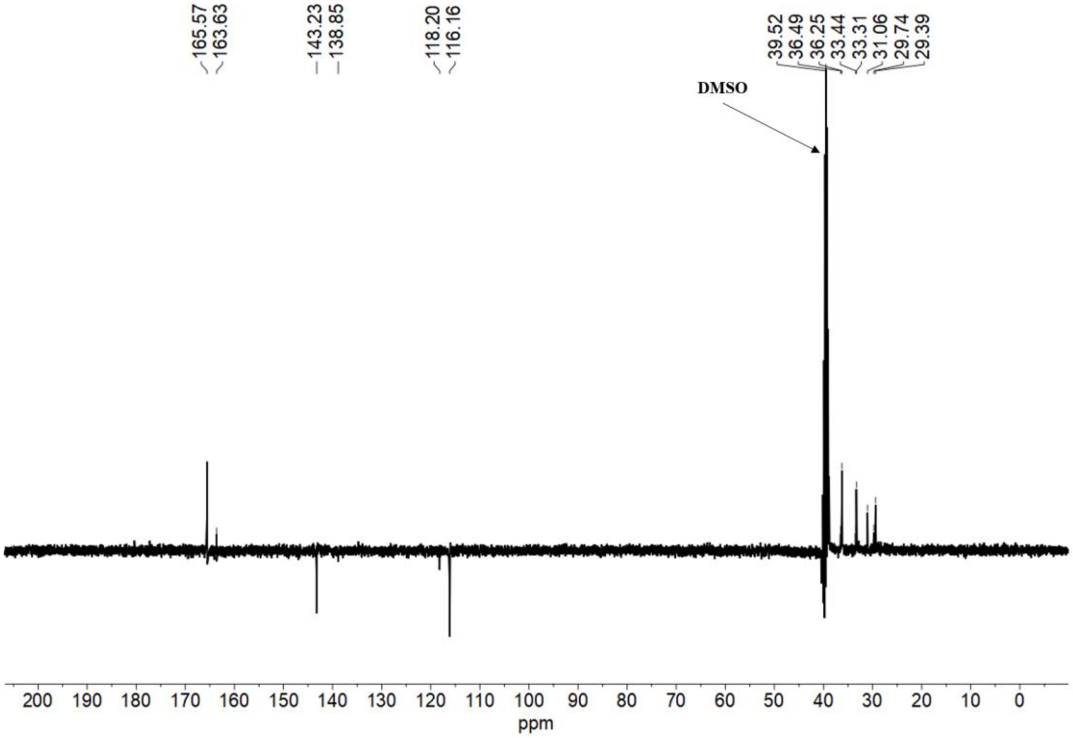

Supplementary Figure 12. C_3A-C_3T 82% *cis* ^{13}C NMR Spectrum – 101 MHz, DMSO-d_6.

Supplementary Note 1. C3A-C3T 82% *cis* ^{1}H NMR and ^{13}C NMR assignments.

C3A-C3T 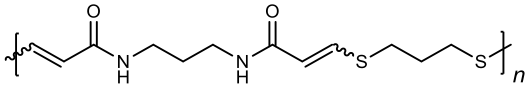

^{1}H NMR (400 MHz, DMSO-d_6) δ 7.85 (t, J = 5.6 Hz, *cis + trans overlapping*, 2H), 7.80 (t, J = 5.3 Hz, *trans*), 7.33 (d, J = 14.9 Hz, *trans*), 6.91 (d, J = 10.0 Hz, *cis* 2H), 5.92 (d, J = 14.7 Hz, *trans*), 5.88 (d, J = 10.0 Hz, *cis* 2H), 3.15 – 3.03 (m, *cis + trans overlapping*, 4H), 2.87 (t, J = 7.4 Hz, *trans*), 2.70 (t, J = 7.3 Hz, *cis* 4H), 1.87 (dt, J = 15.0, 7.6 Hz, 2H), 1.54 (m, 2H). ^{13}C NMR (101 MHz, DMSO-d_6) δ 165.57$_{cis}$, 163.63$_{trans}$, 143.23$_{cis}$, 138.85$_{trans}$, 118.20$_{trans}$, 116.16$_{cis}$, 36.49$_{trans}$, 36.25$_{cis}$, 33.44$_{trans}$, 33.31$_{cis}$, 31.05, 29.74$_{trans}$, 29.39$_{cis}$.

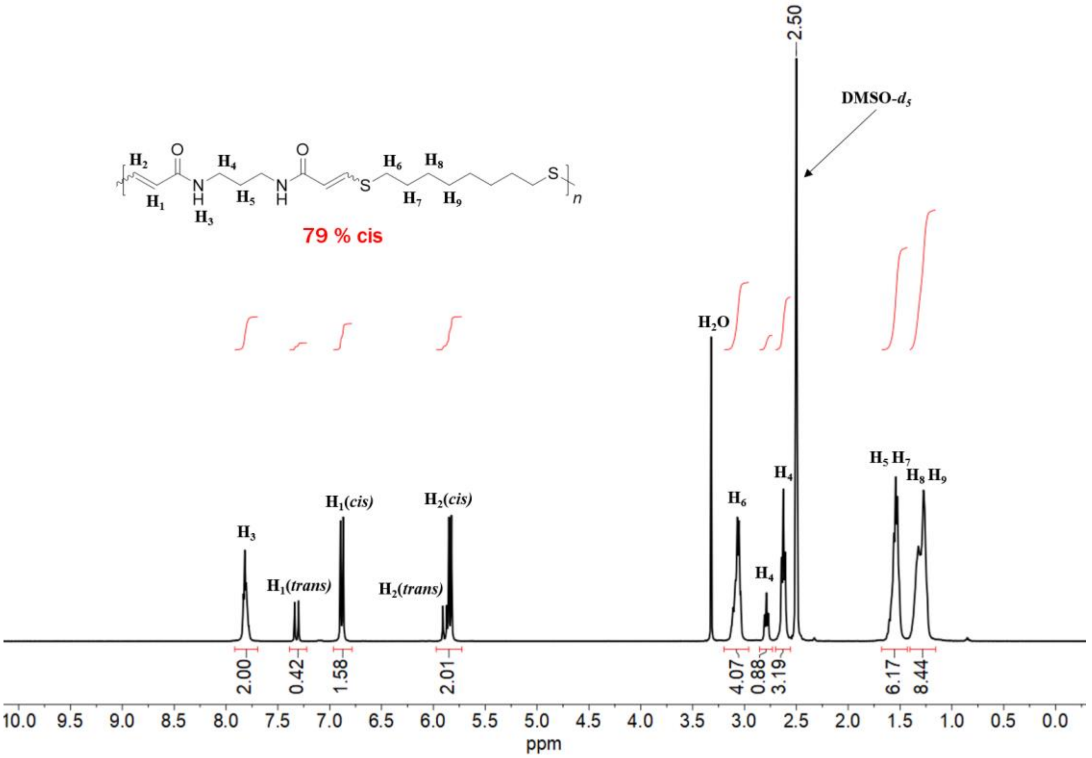

Supplementary Figure 13. C3A-C8T 79% *cis* ^{1}H NMR Spectrum – 400 MHz, DMSO-d_6.

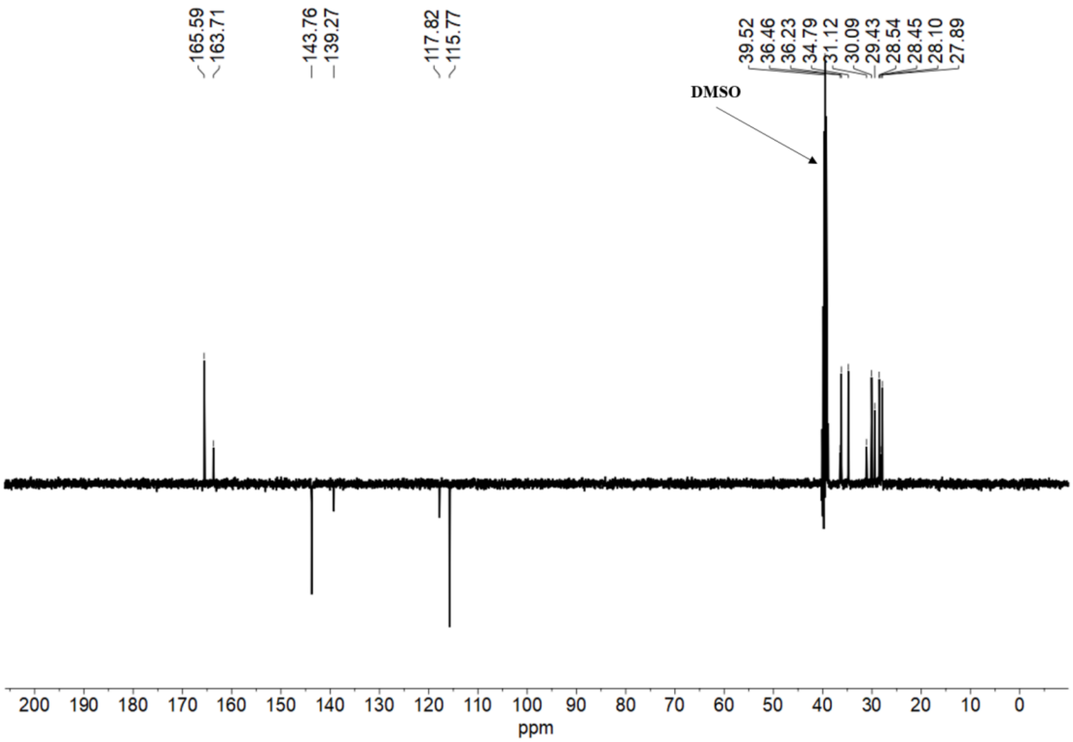

Supplementary Figure 14. C_3A-C_8T 79% *cis* ^{13}C NMR Spectrum – 101 MHz, DMSO-d_6.

Supplementary Note 2. C_3A-C_8T 79% *cis* ^1H NMR and ^{13}C NMR assignments.

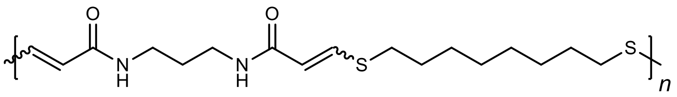

C_3A-C_8T

^1H NMR (400 MHz, DMSO-d_6) δ 7.82 (t, J = 5.6 Hz, *cis + trans overlapping* 2H), 7.32 (d, J = 15.0 Hz, *trans*), 6.88 (d, J = 10.1 Hz, 2H), 5.89 (d, J = 15.0 Hz, *trans*), 5.84 (d, J = 10.0 Hz, *cis*, 2H), 3.16 – 3.00 (m, *cis + trans overlapping*, 4H), 2.79 (t, J = 7.3 Hz, *trans*), 2.62 (t, J = 7.3 Hz, *cis*, 4H), 1.63 – 1.48 (m, 6H), 1.42 – 1.20 (m, 8H). ^{13}C NMR (101 MHz, DMSO-d_6) δ 165.59$_{cis}$, 163.71$_{trans}$, 143.76$_{cis}$, 139.27$_{trans}$, 117.82$_{trans}$, 115.77$_{cis}$, 36.46$_{trans}$, 36.23$_{cis}$, 34.79, 31.12$_{trans}$, 30.09$_{cis}$, 29.43$_{cis}$, 28.54, 28.45$_{trans}$, 28.10$_{trans}$, 27.89$_{cis}$.

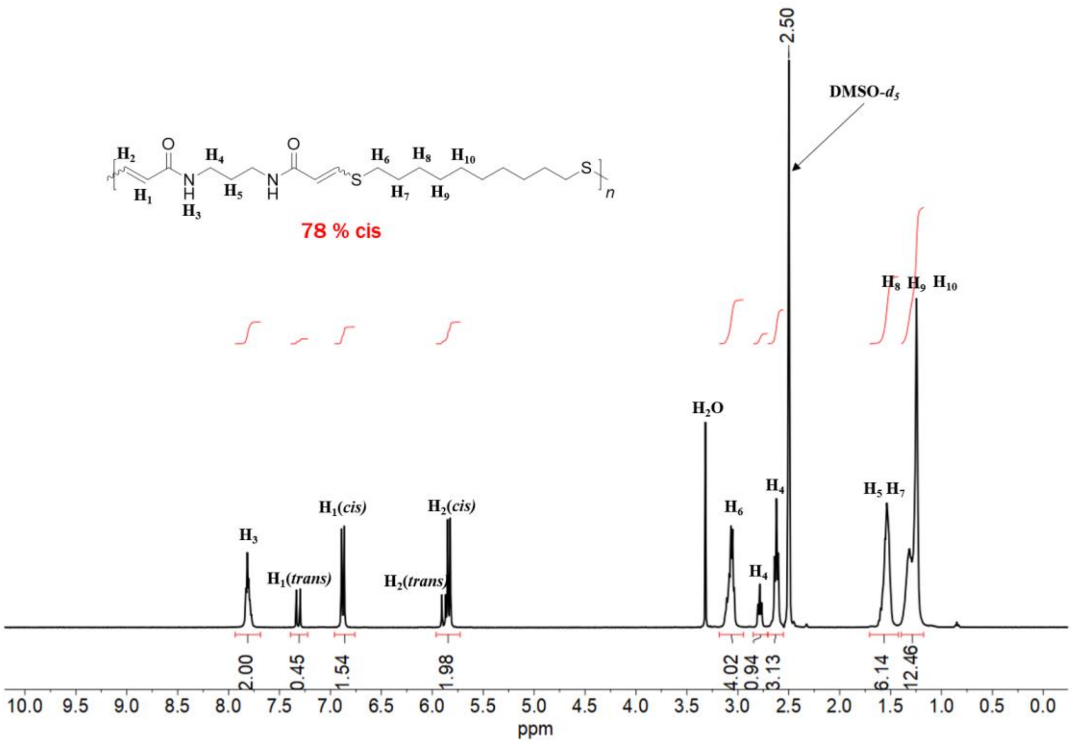

Supplementary Figure 15. C3A-C10T 78% *cis* ^{1}H NMR Spectrum – 400 MHz, DMSO-d_6.

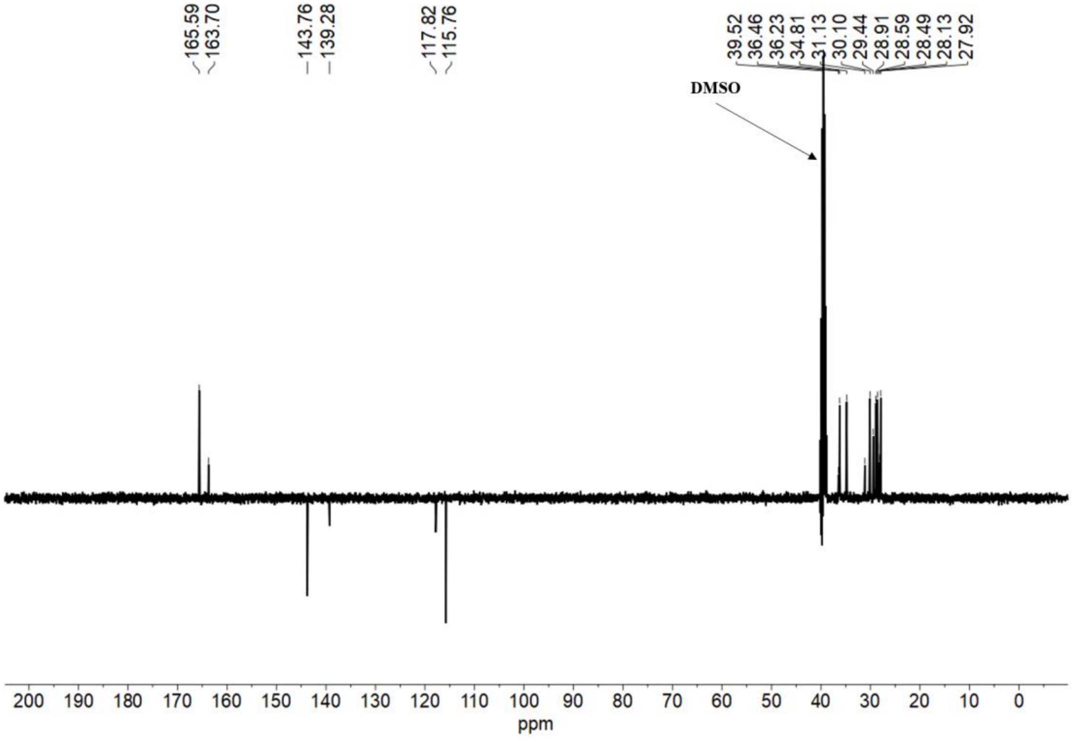

Supplementary Figure 16. C3A-C10T 78% *cis* ^{13}C NMR Spectrum – 101 MHz, DMSO-d_6.

Supplementary Note 3. C_3A-$C_{10}T$ 78% *cis* ^1H NMR and ^{13}C NMR assignments.

C_3A-$C_{10}T$

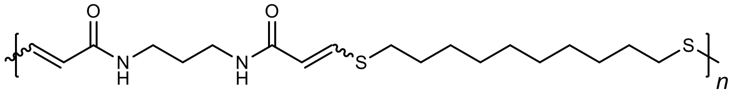

^1H NMR (400 MHz, DMSO-d_6) δ 7.82 (t, J = 5.6 Hz, *cis* + *trans overlapping*, 2H), 7.32 (d, J = 15.0 Hz, *trans*), 6.88 (d, J = 10.1 Hz, *cis*, 2H), 5.89 (d, J = 15.0 Hz, *trans*), 5.84 (d, J = 10.0 Hz, *cis*, 2H), 3.17 – 2.99 (m, *cis* + *trans overlapping*, 4H), 2.78 (t, J = 7.3 Hz, *trans*), 2.62 (t, J = 7.3 Hz, *cis,* 4H), 1.63 – 1.44 (m, 6H), 1.40 – 1.17 (m, 12H). ^{13}C NMR (101 MHz, DMSO-d_6) δ 165.59_{cis}, 163.70_{trans}, 143.76_{cis}, 139.28_{trans}, 117.82_{trans}, 115.76_{cis}, 36.46_{trans}, 36.23_{cis}, 34.81, 31.13_{trans}, 30.10_{cis}, 29.44_{cis}, 28.91, 28.59, 28.51_{trans}, 28.13_{trans}, 27.92_{cis}.

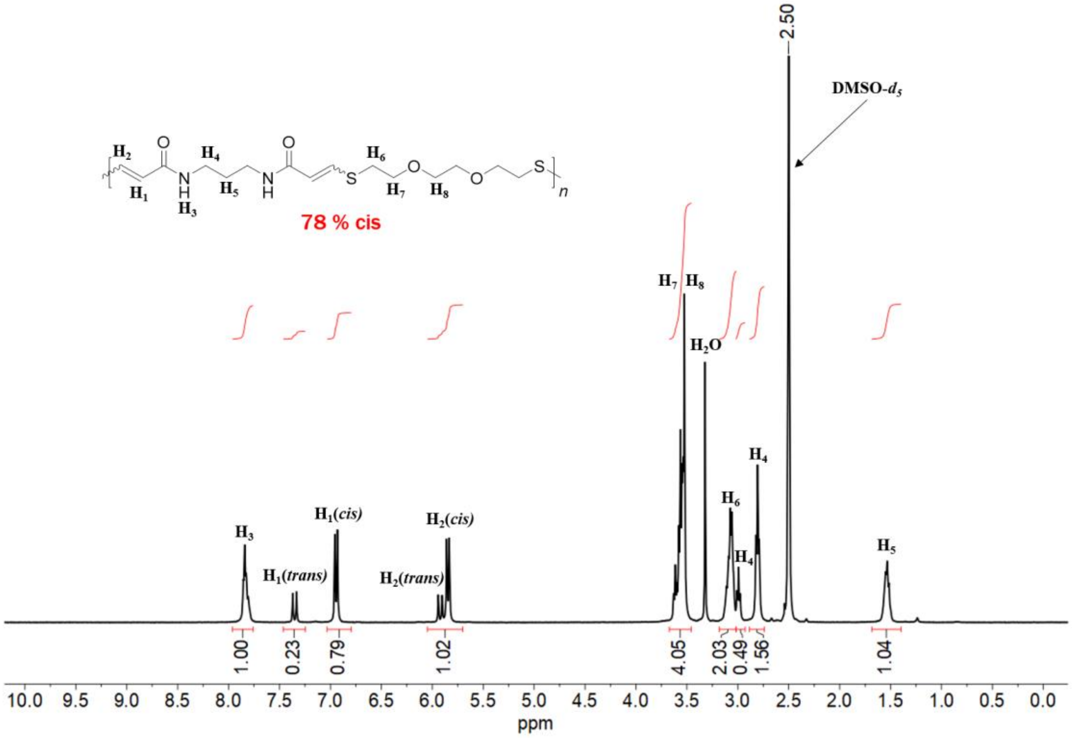

Supplementary Figure 17. C_3A-$C_{DEG}T$ 78% *cis* ^1H NMR Spectrum – 400 MHz, DMSO-d_6.

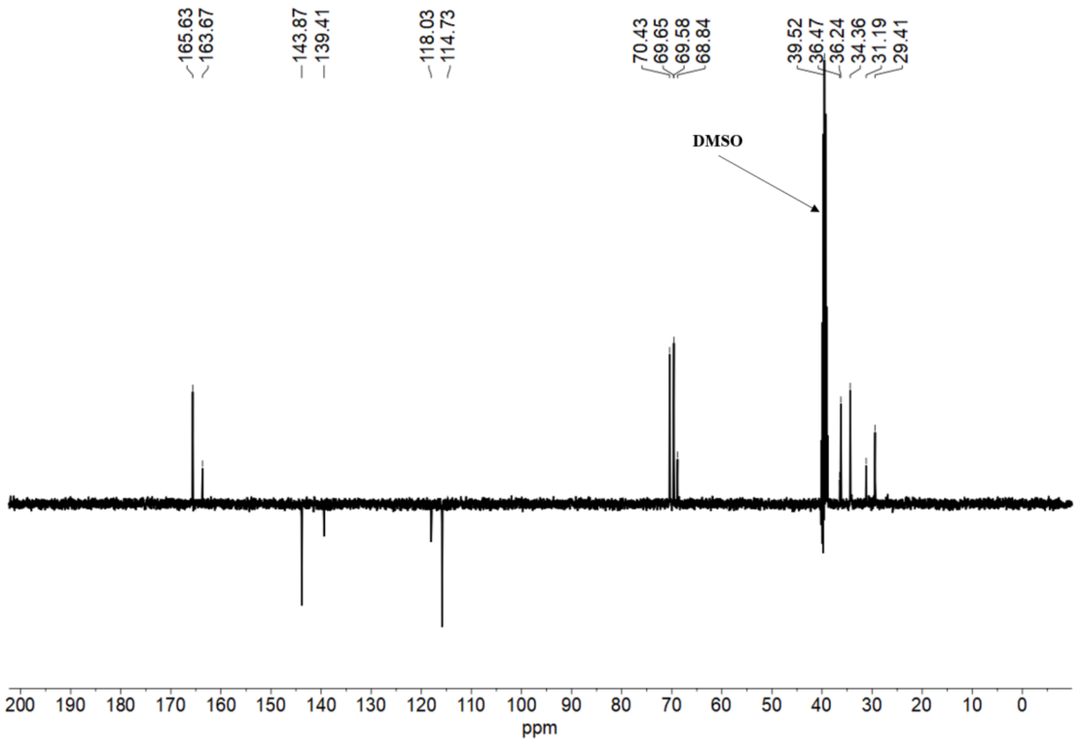

Supplementary Figure 18. C_3A-$C_{DEG}T$ 78% *cis* ^{13}C NMR Spectrum – 101 MHz, DMSO-d_6.

Supplementary Note 4. C_3A-$C_{DEG}T$ 78% *cis* ^{1}H NMR and ^{13}C NMR assignments.

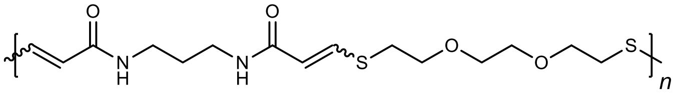

C_3A-$C_{DEG}T$

^{1}H NMR (400 MHz, DMSO-d_6) δ 7.84 (t, J = 5.9 Hz, *cis + trans overlapping*, 2H), 7.35 (d, J = 15.0 Hz, *trans*), 6.94 (d, J = 10.0 Hz, *cis*, 2H), 5.92 (d, J = 15.0 Hz, *trans*), 5.84 (d, J = 10.1, *cis*, 2H), 3.64 – 3.48 (m, *cis + trans overlapping*, 8H), 3.07 (m, *cis + trans overlapping*, 4H), 2.99 (t, J = 6.4 Hz, *trans*), 2.81 (t, J = 6.7 Hz, *cis*, 4H), 1.58 – 1.49 (m, 2H). ^{13}C NMR (101 MHz, DMSO-d_6) δ 165.63_{cis}, 163.67_{trans}, 143.87_{cis}, 139.41_{trans}, 118.03_{trans}, 114.73_{cis}, 70.43_{cis}, 69.65_{trans}, 69.58_{cis}, 68.84_{trans}, 36.47_{trans}, 36.24_{cis}, 34.36, 31.19_{trans}, 29.41_{cis}.

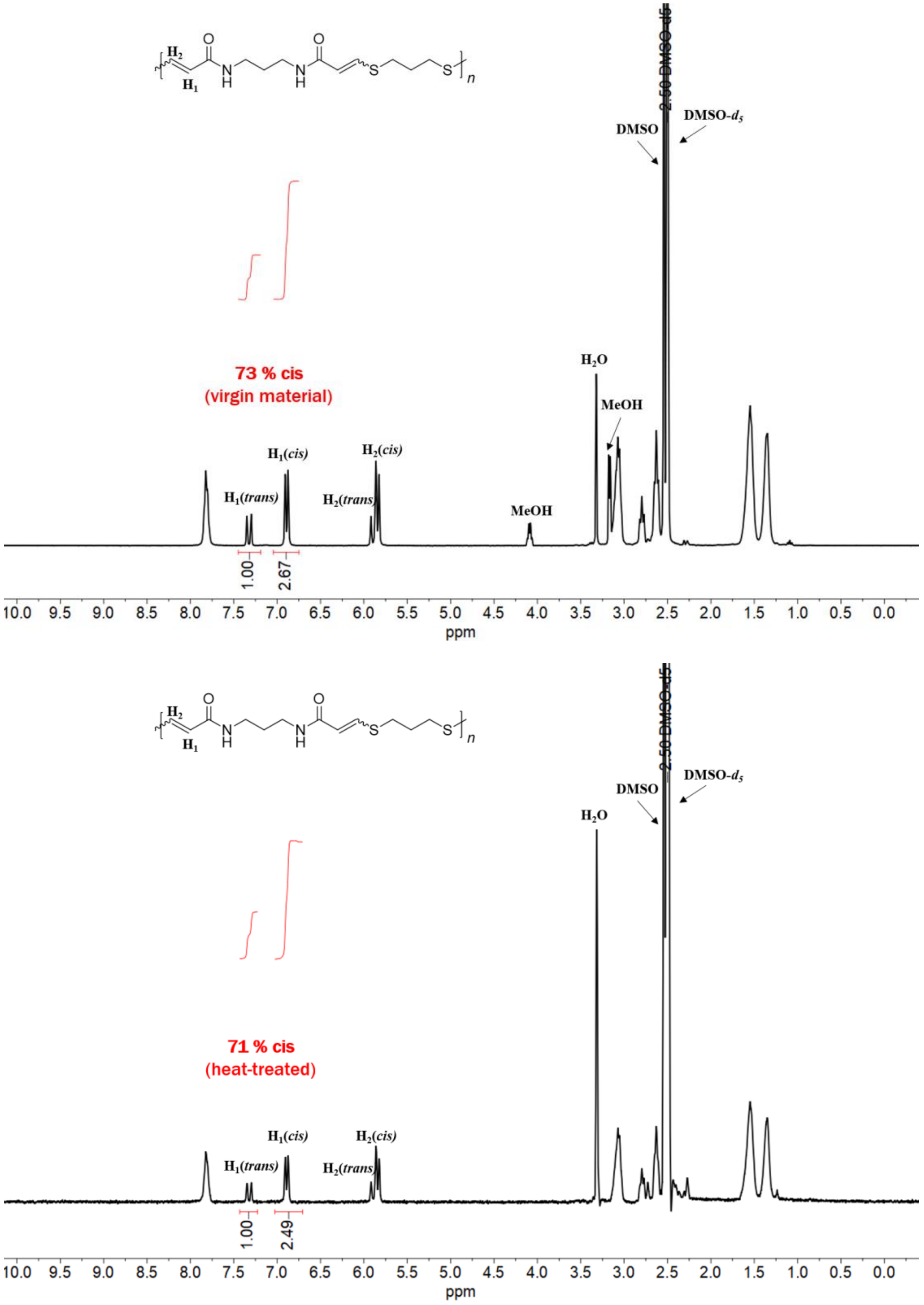

Supplementary Figure 19. ^{1}H NMR spectroscopy (400 MHz, DMSO-d_6) of as-synthesized & heat treated C_3A-C_6T indicating minimal isomerization of double bond. Top ─ as-synthesized polymer. Bottom ─ Polymer heated under N_2 atmosphere at 150° C, 12 h.

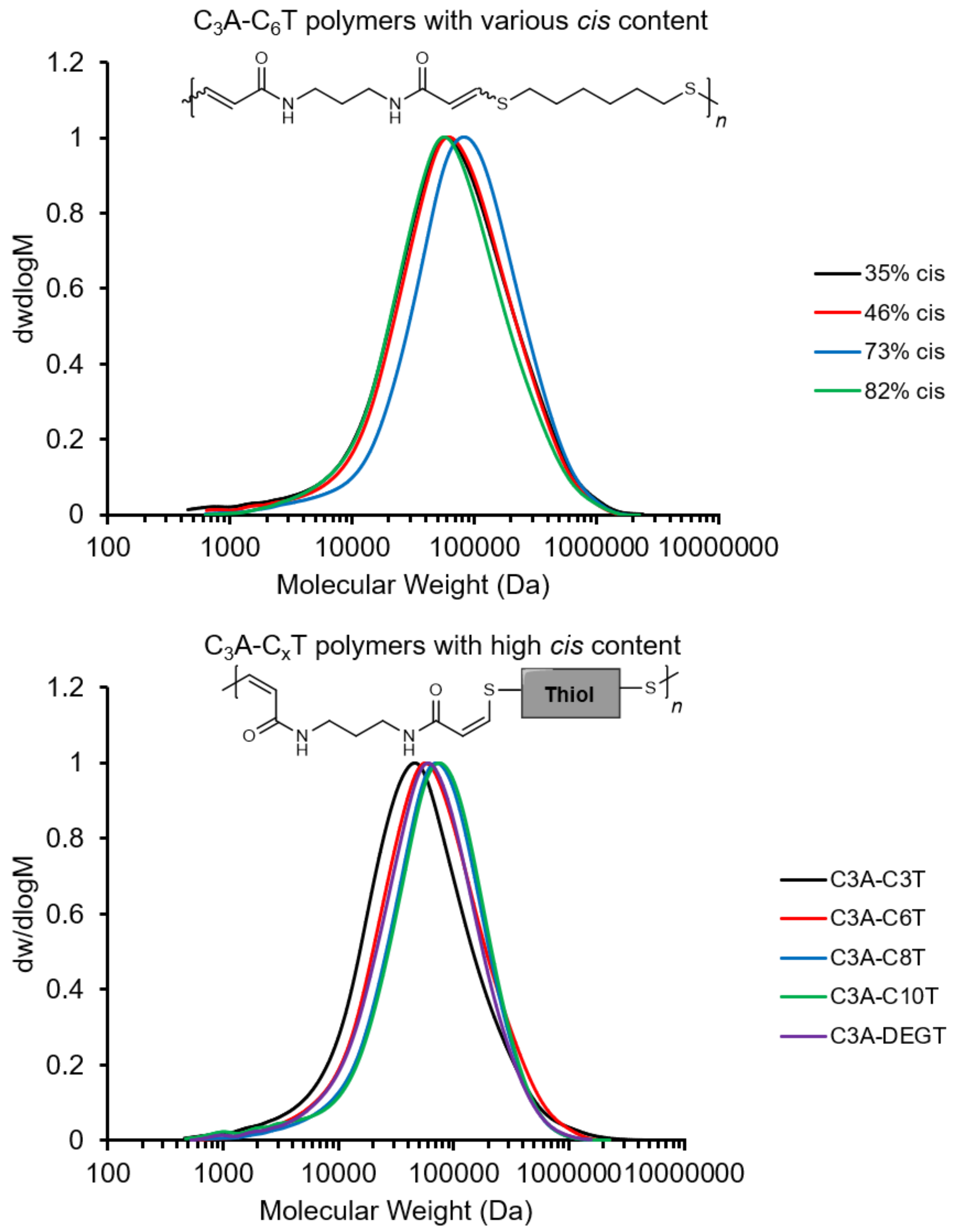

Supplementary Figure 20. Normalised size exclusion chromatogram of polyamides. Top – C_3A-C_6T variable *cis* content. Bottom – C_3A with various thiol comonomers at high *cis* content.

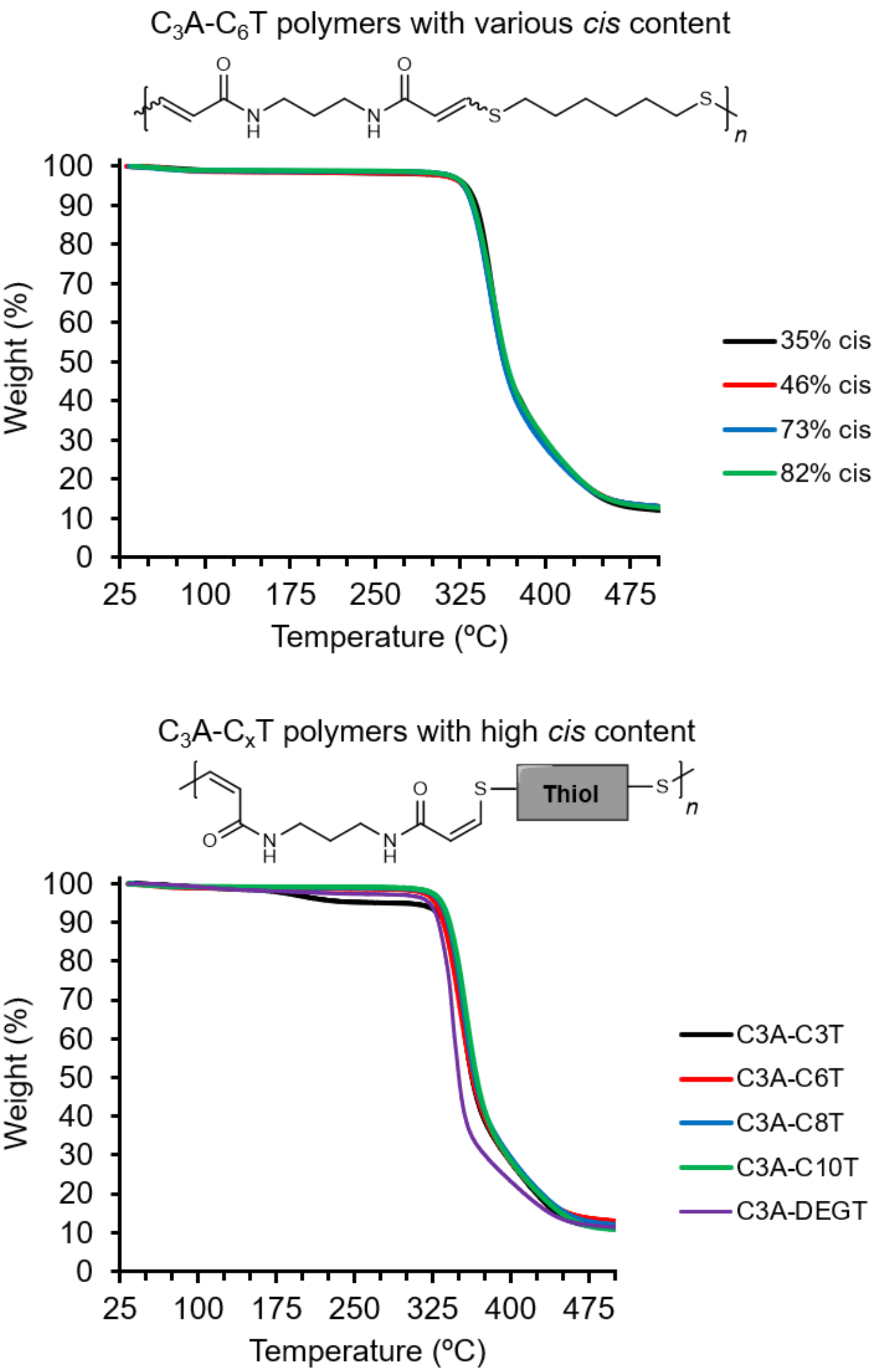

Supplementary Figure 21. TGA thermograms of polyamides. Top – C_3A-C_6T variable *cis* content. Bottom – C_3A with various thiol comonomers at high *cis* content.

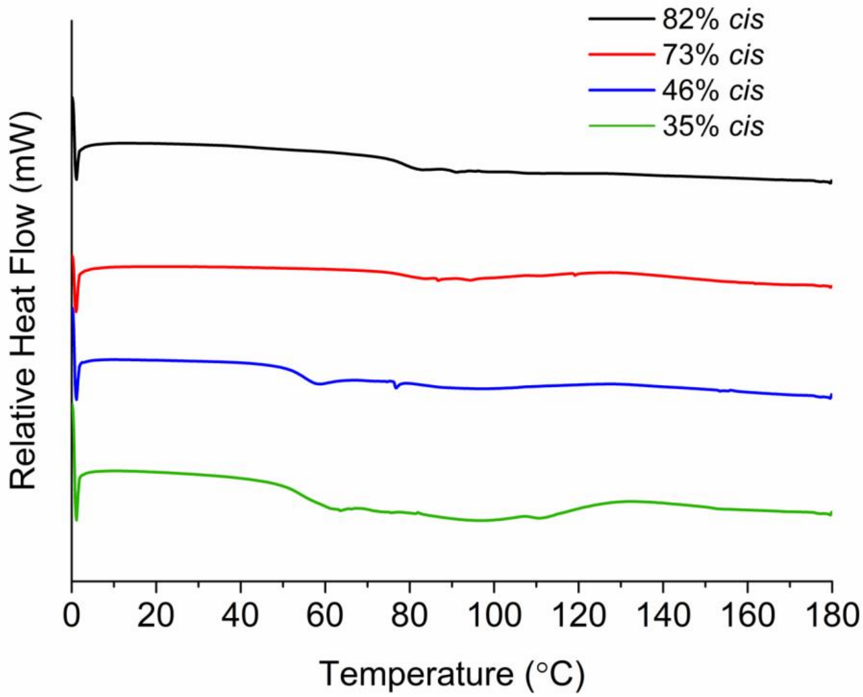

Supplementary Figure 22. DSC thermograms of 1st heating cycle for C_3A-C_6T with variable *cis* content.

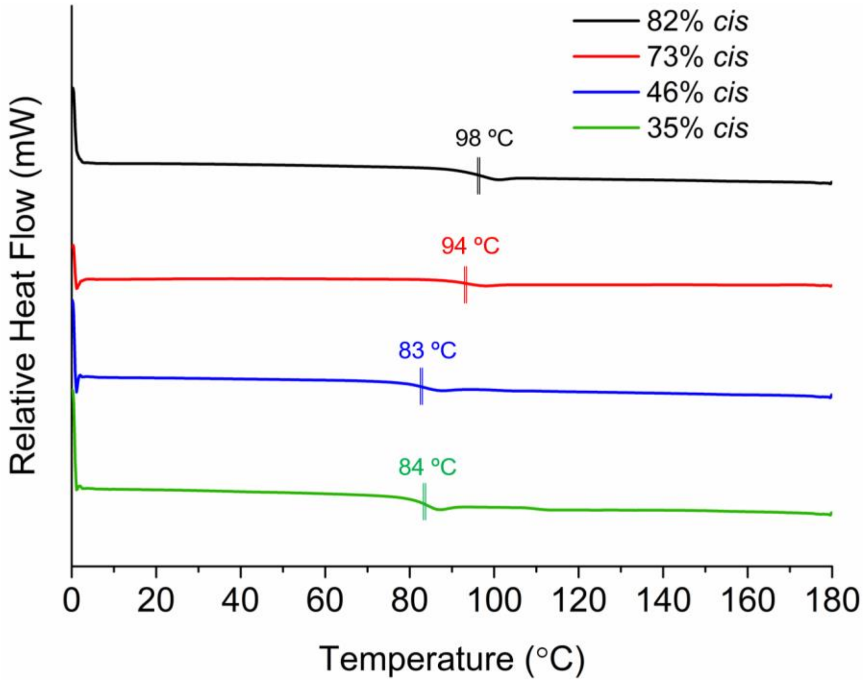

Supplementary Figure 23. DSC thermograms of 2nd heating cycle for C_3A-C_6T with variable *cis* content. Approximate position of T_g is indicated by vertical hashmark.

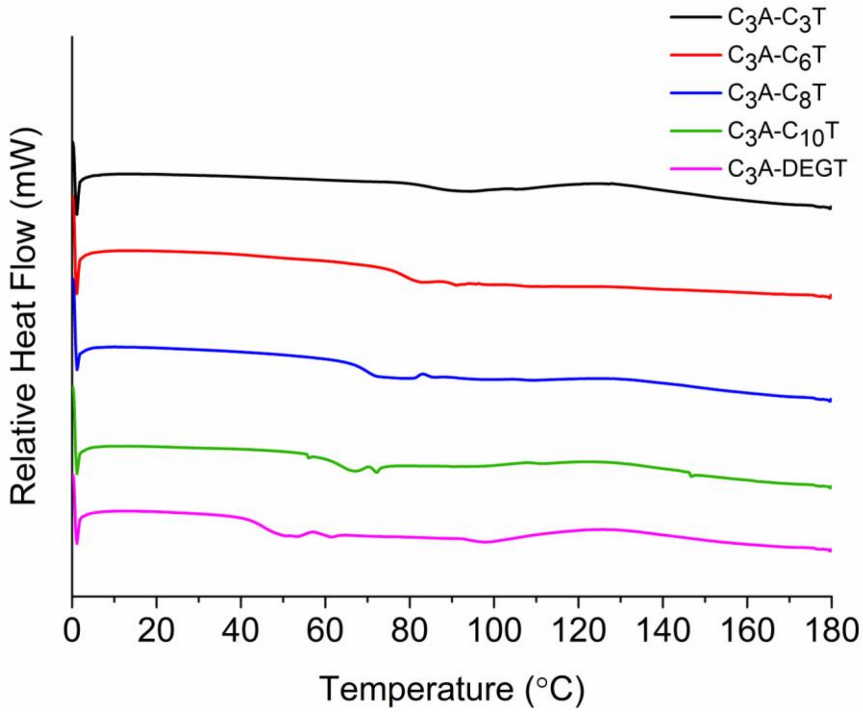

Supplementary Figure 24. DSC thermograms of 1st heating cycle for polyamides with high *cis* content.

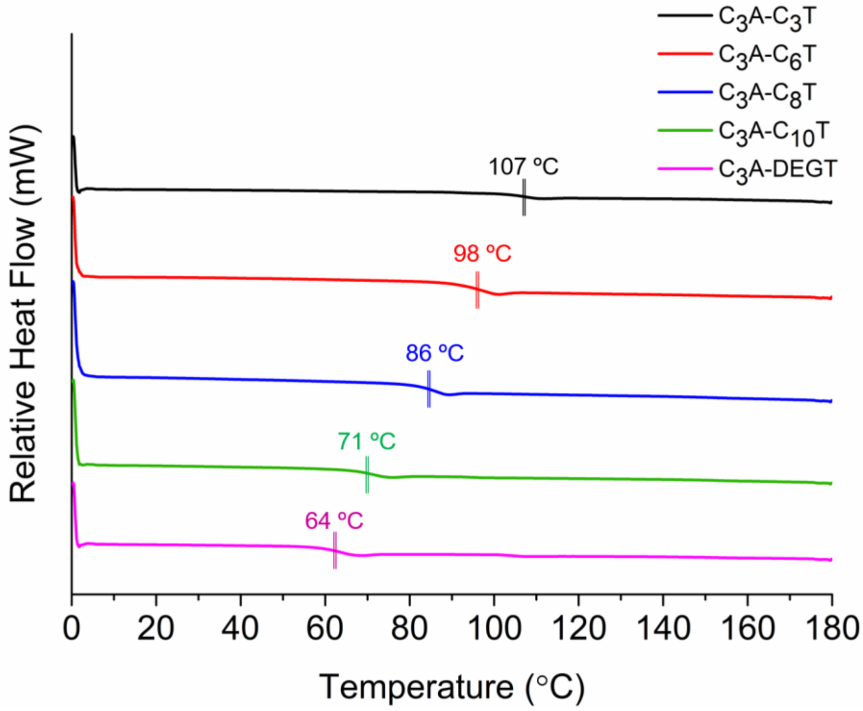

Supplementary Figure 25. DSC thermograms of 2nd heating cycle for polyamides with high *cis* content. Approximate position of T_g is indicated by vertical hashmark.

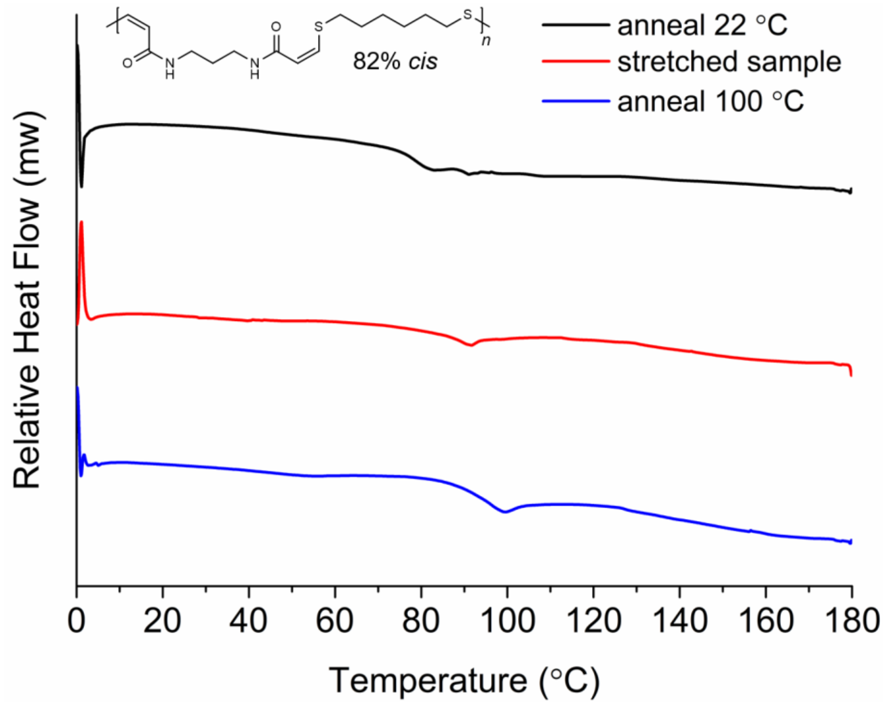

Supplementary Figure 26. DSC Thermograms of 1st heating cycle for three separate samples. Top ─ C_3A-C_6T 82% *cis*. Middle ─ after stretching sample to break (uniaxial tensile testing). Bottom ─ annealing for 12 h above the glass transition temperature of the polymer. No melt transitions were detected in any sample.

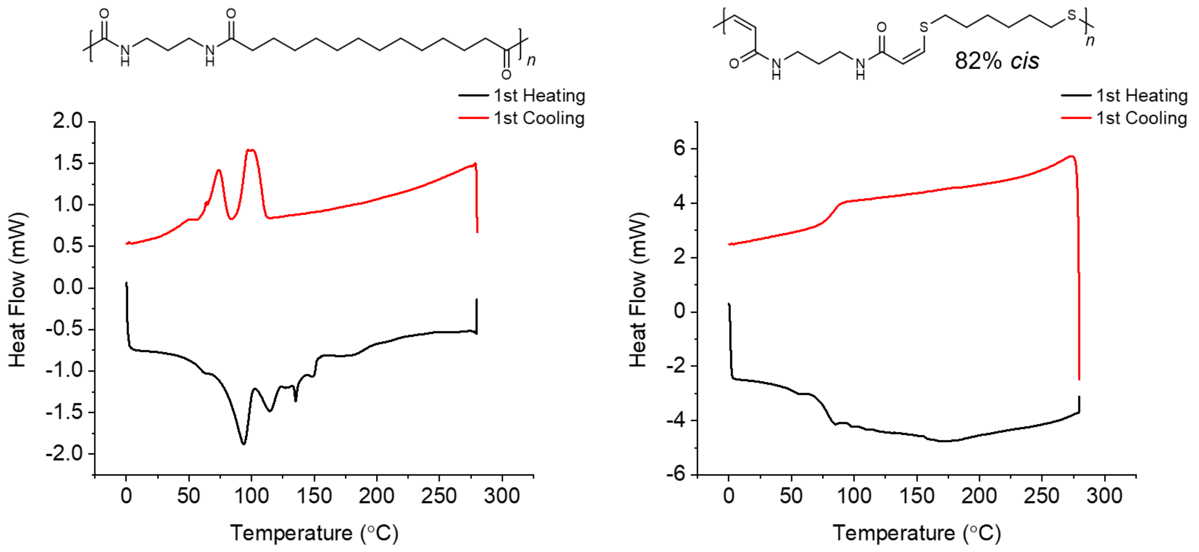

Supplementary Figure 27. DSC Thermograms of 1st heating/cooling cycle for Left ─ Nylon 3,14 and Right ─ C_3A-C_6T 82% *cis*. Nylon 3,14 was tested as-synthesised from the melt polycondensation of 1,3-diaminopropane and tetradecanedicarboxylic acid (200 °C, 200 mTorr vacuum).

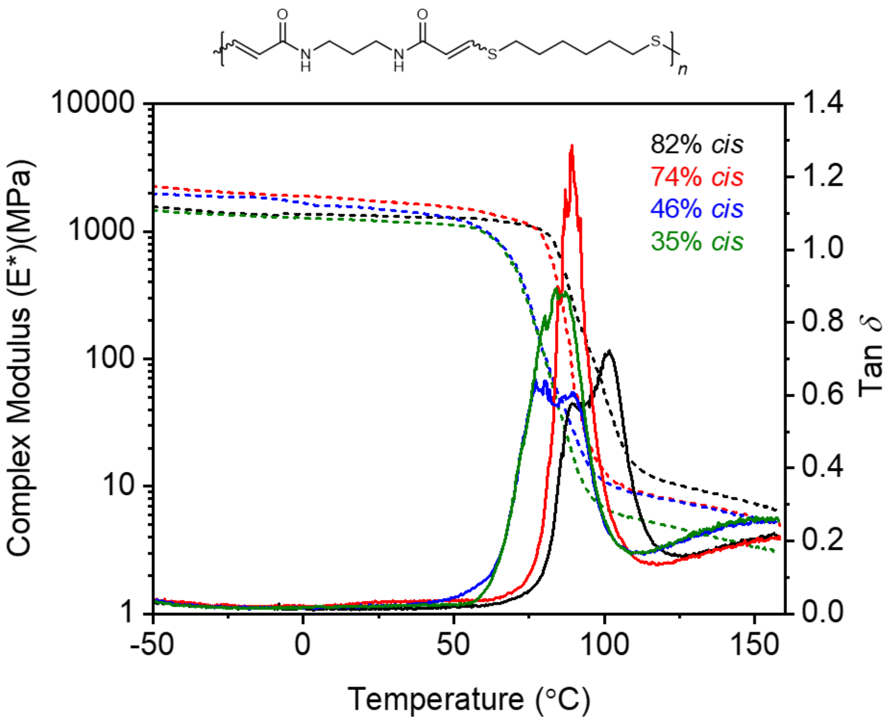

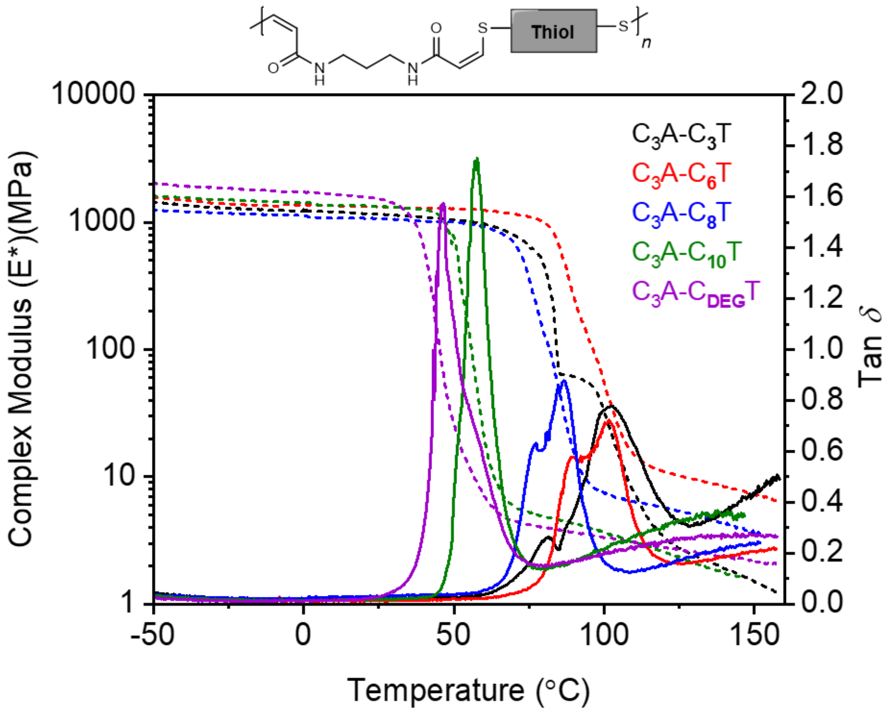

Supplementary Figure 28. Representative DMA for polyamides. Top ─ C_3A-C_6T with variable stereochemistry. Bottom ─ C_3A with different thiols and high *cis* content.

Supplementary Table 1. Shape memory behavior of C_3A-C_6T polymer samples with variable stereochemistry, examined using DMA. (n = 3)

Sample	Strain Fixation	Strain Recovery (%)	Recovery Temperature (°C)
C_3A-$C_6T_{(37\%\ cis)}$	99 ± 2	100	34 ± 3
C_3A-$C_6T_{(46\%\ cis)}$	98 ± 4	100	35 ± 4
C_3A-$C_6T_{(73\%\ cis)}$	99 ± 1	100	60 ± 2
C_3A-$C_6T_{(82\%\ cis)}$	99 ± 2	100	61 ± 2

Supplementary Table 2. Shape memory behavior of C_3A-C_xT polymer samples with various thiols and high *cis* content, examined using DMA. (n = 3)

Sample	Strain Fixation	Strain Recovery (%)	Recovery Temperature (°C)
C_3A-$C_3T_{(82\%\ cis)}$	99 ± 1	100	61 ± 1
C_3A-$C_6T_{(82\%\ cis)}$	99 ± 2	100	61 ± 2
C_3A-$C_8T_{(79\%\ cis)}$	99 ± 1	100	61 ± 3
C_3A-$C_{10}T_{(77\%\ cis)}$	99 ± 2	100	45 ± 1
C_3A-$C_{DEG}T_{(78\%\ cis)}$	99 ± 1	100	35 ± 5

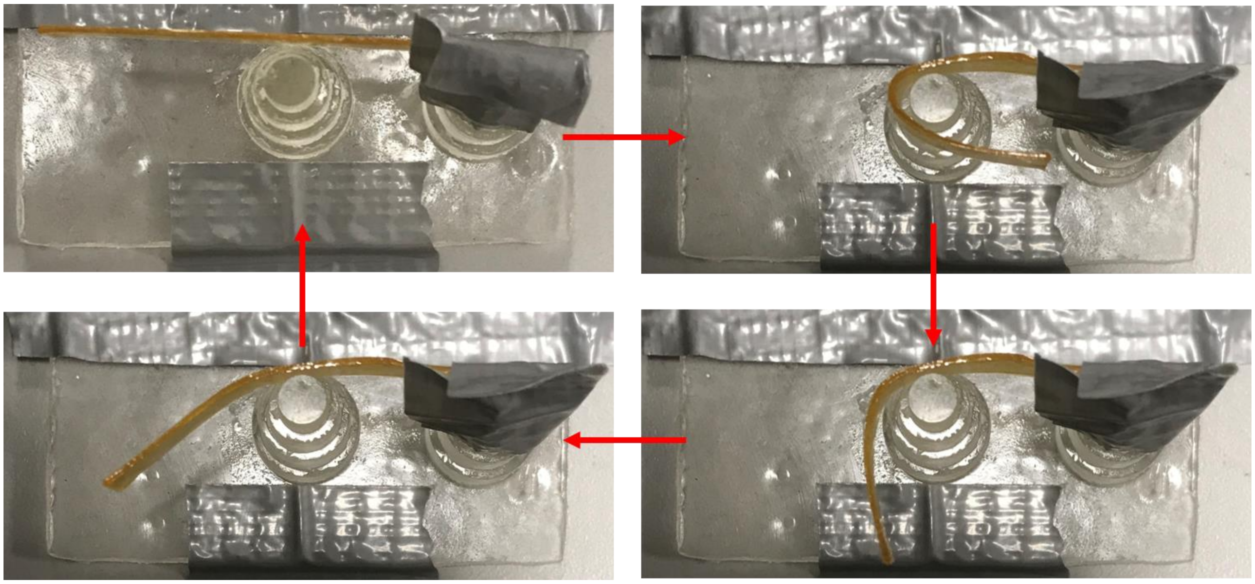

Supplementary Figure 29. Experimental set-up for cyclic shape memory testing with 1 cycle shown for reference.

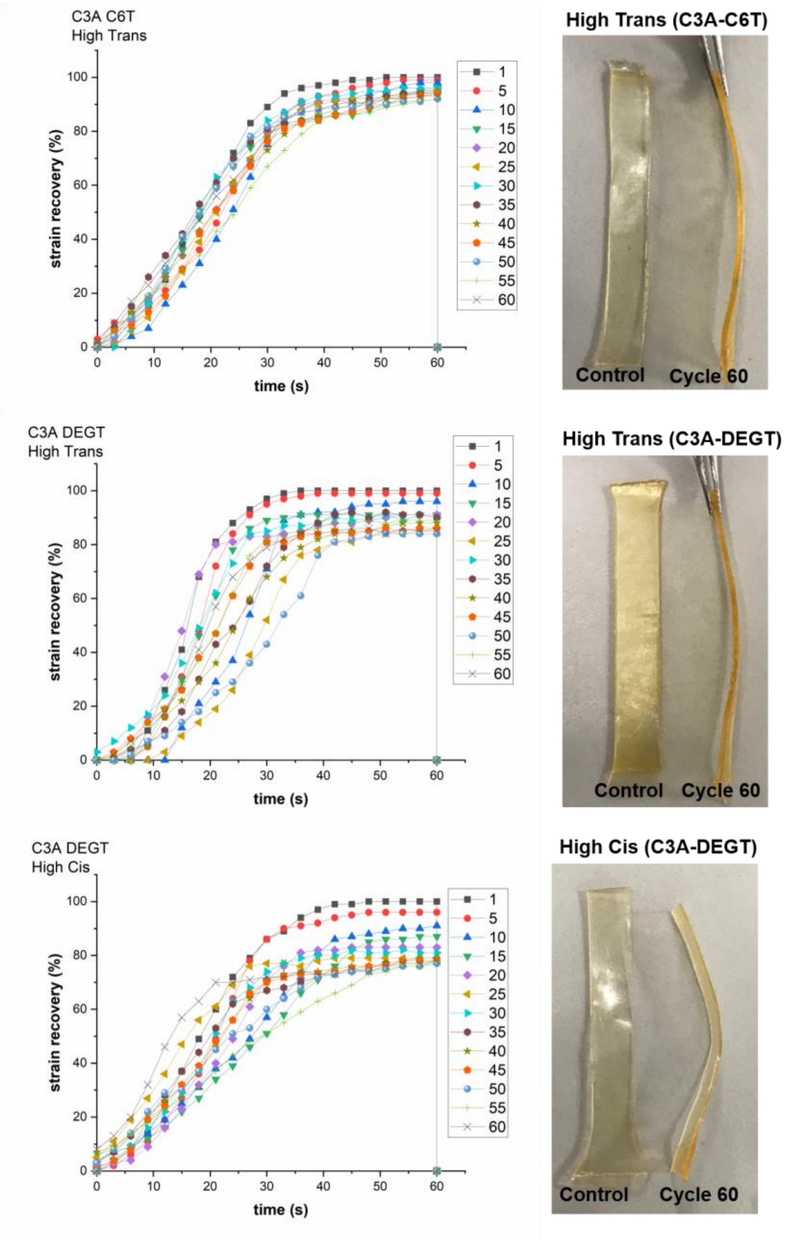

Supplementary Figure 30. Cyclic shape memory (60 cycles) for various polyamides. The data is plotted every 5 cycles for clarity.

Supplementary Table 3. Strain recovery of polyamides for cyclic shape memory. (60 cycles, n = 3)

**Cycle**	C_3A-C_6T high *trans*	C_3A-$C_{DEG}T$ high *trans*	C_3A-$C_{DEG}T$ high *cis*
1	100 ± 0	99 ± 1	100 ± 0
20	95 ± 2	91 ± 5	83 ± 6
40	95 ± 4	88 ± 3	79 ± 4
60	93 ± 3	85 ± 4	78 ± 5

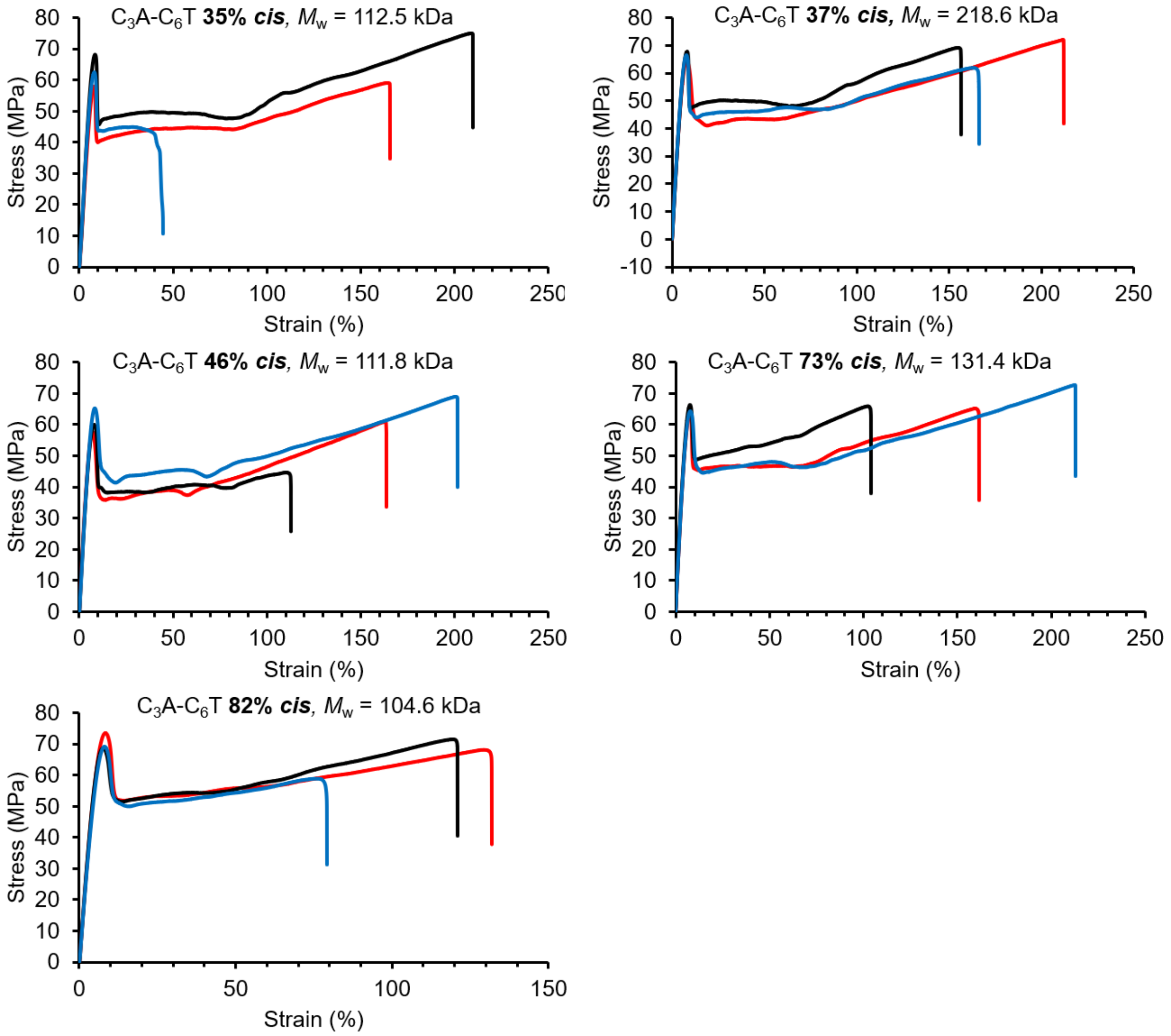

Supplementary Figure 31. Stress *vs* strain curves of C_3A-C_6T polymers with variable *cis* content tested at 10 mm min^{-1} strain rate. Summary of results are shown in Supplementary Table 4.

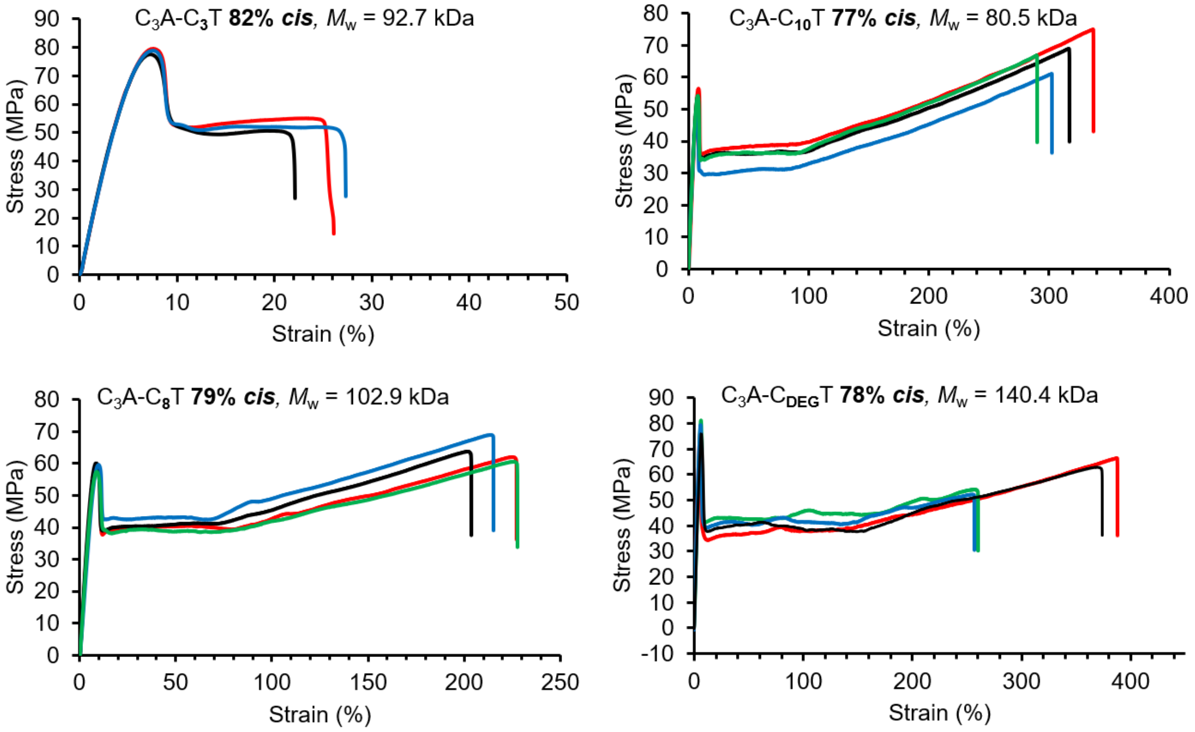

Supplementary Figure 32. Stress *vs* strain curves of C3A-CxT polymers (thiol variation) with high *cis* content tested at 10 mm min^{-1} strain rate. Summary of results are shown in Supplementary Table 4.

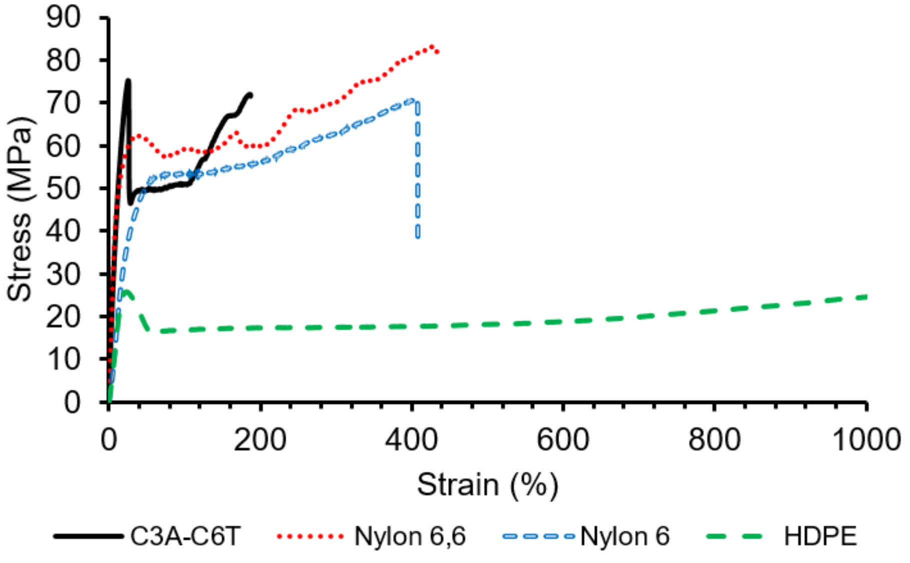

Supplementary Figure 33. Representative stress *vs* strain curves of C3A-C6T 82% *cis*, Nylon 6, Nylon 6,6, and HDPE tested at 22 °C at 10 mm min^{-1} strain rate. (n ≥ 2)

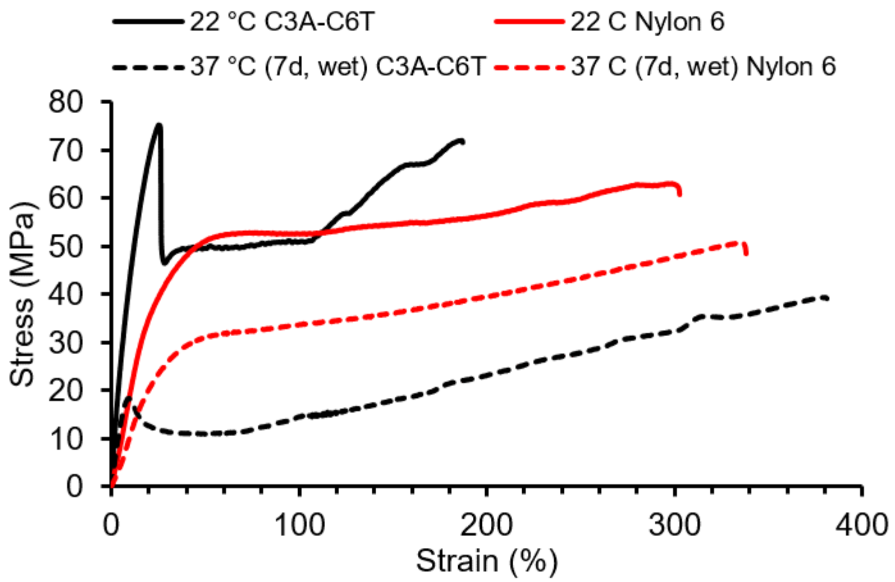

Supplementary Figure 34. Stress *vs* strain curves of C_3A-C_6T 82% *cis*, and Nylon 6 tested at 22 °C and after immersion in PBS solution for 7 d at 37 °C. Testing was performed at 37 °C in order to mimic biological conditions and at 10 mm min^{-1} strain rate. (n = 1 for each sample)

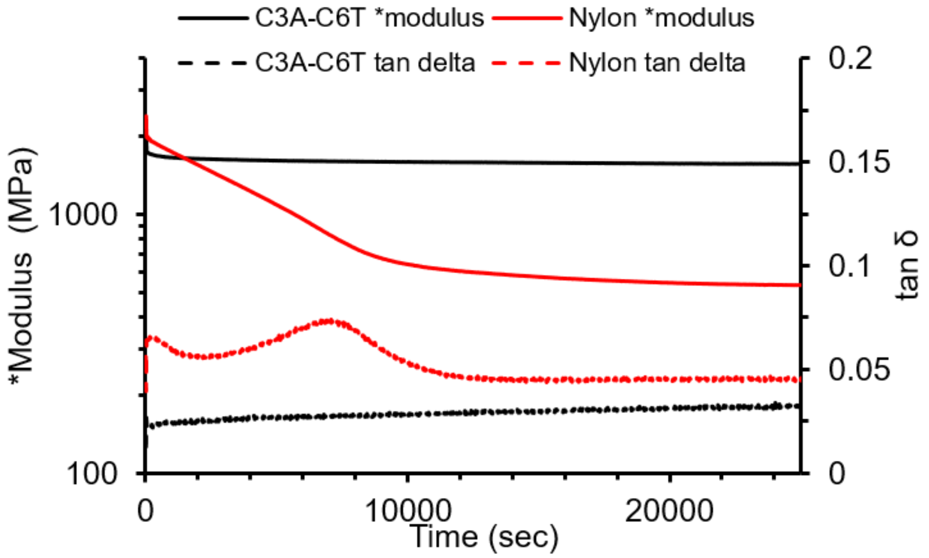

Supplementary Figure 35. Representative DMA of C_3A-C_6T 82% *cis* and Nylon 6 immersed in water at 37 °C. (n = 3 for each sample)

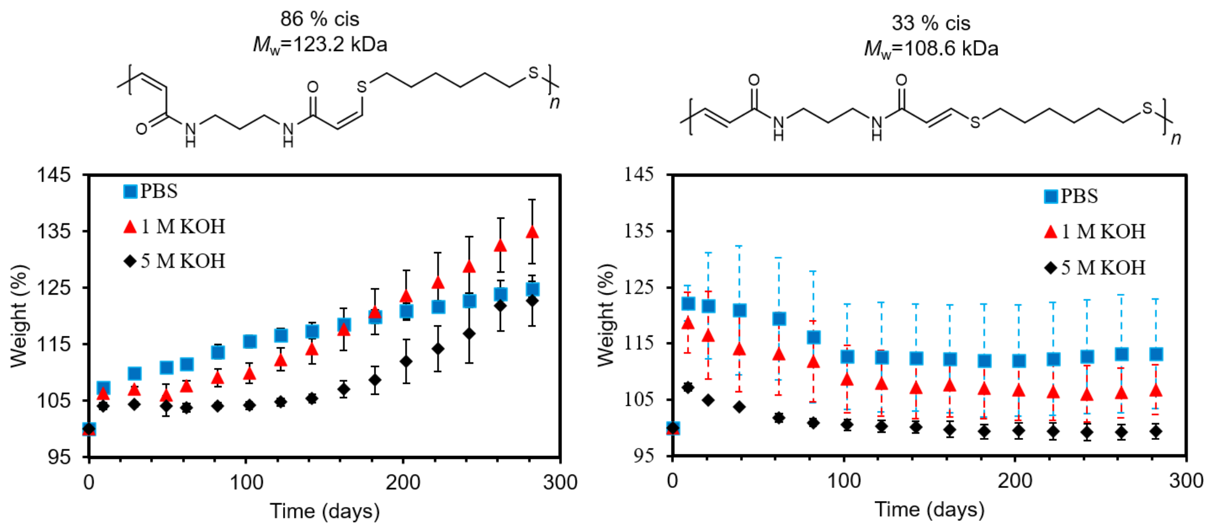

Supplementary Figure 36. Degradation experiments for C_3A-C_6T polymers (high *cis* & high *trans)* by aqueous immersion at 37 °C with periodic weight measurements. Error bars represent 1 s.d. (n = 3 for each polymer in each solution)

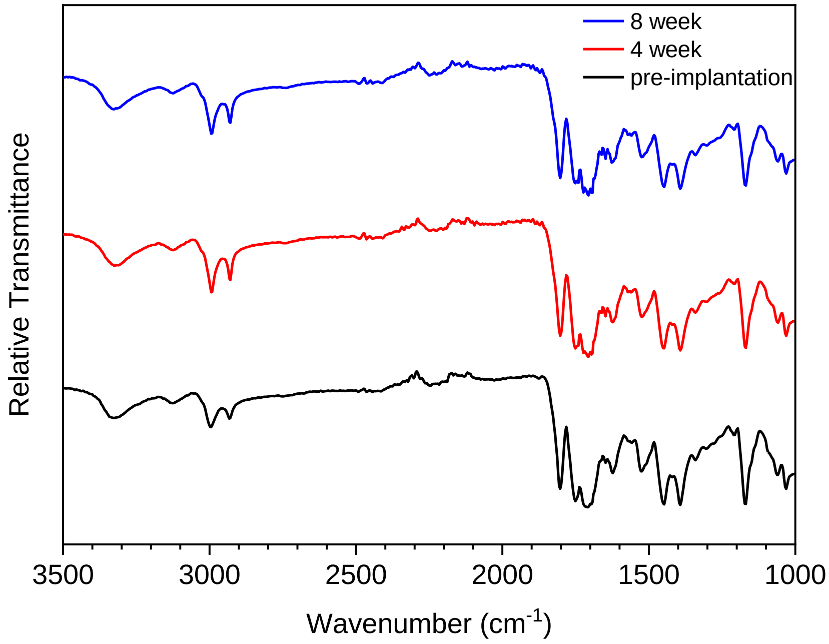

Supplementary Figure 37. IR spectra for C_3A-C_6T (82% *cis*) before implantation, 4-week implantation, and 8-week implantation.

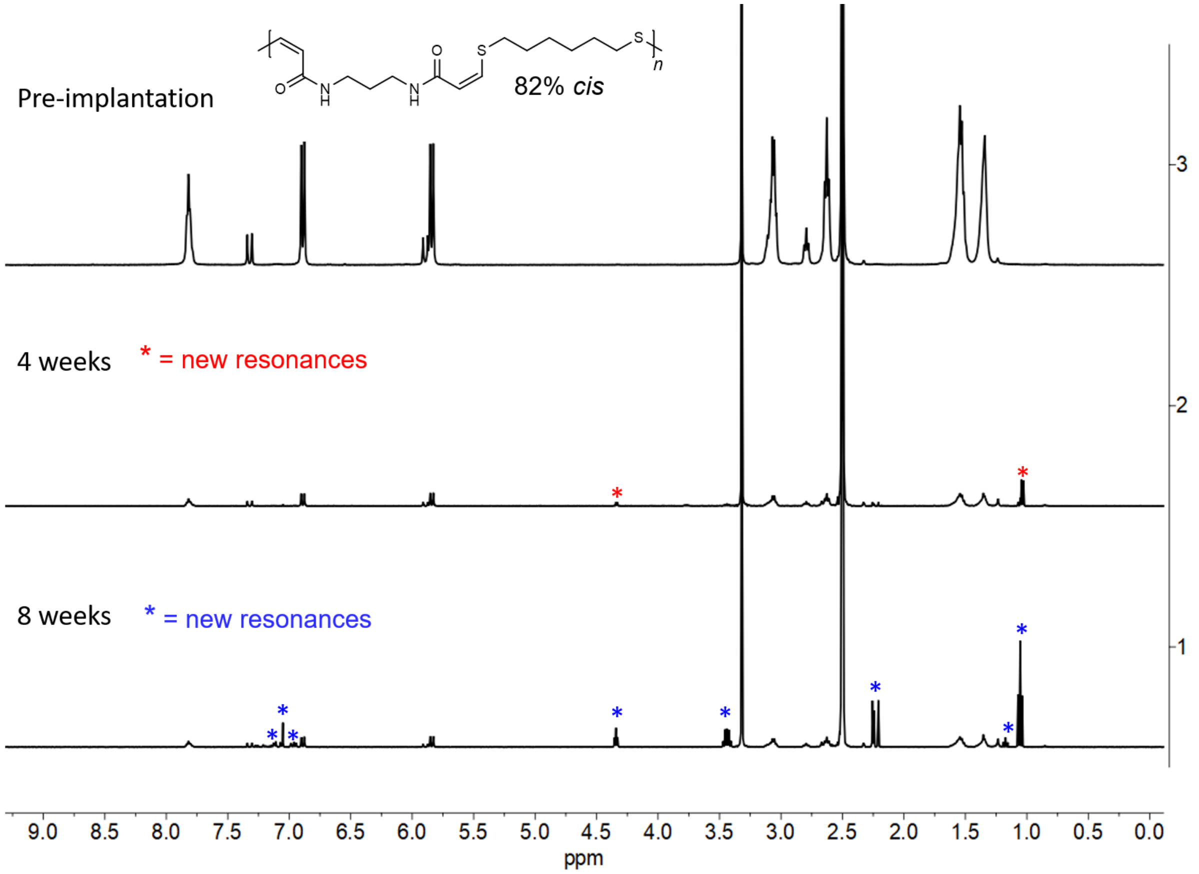

Supplementary Figure 38. ^{1}H NMR spectra for C_3A-C_6T (82% *cis*) before implantation, 4-week implantation, and 8-week implantation. New resonances are indicated with (*) for each time point.

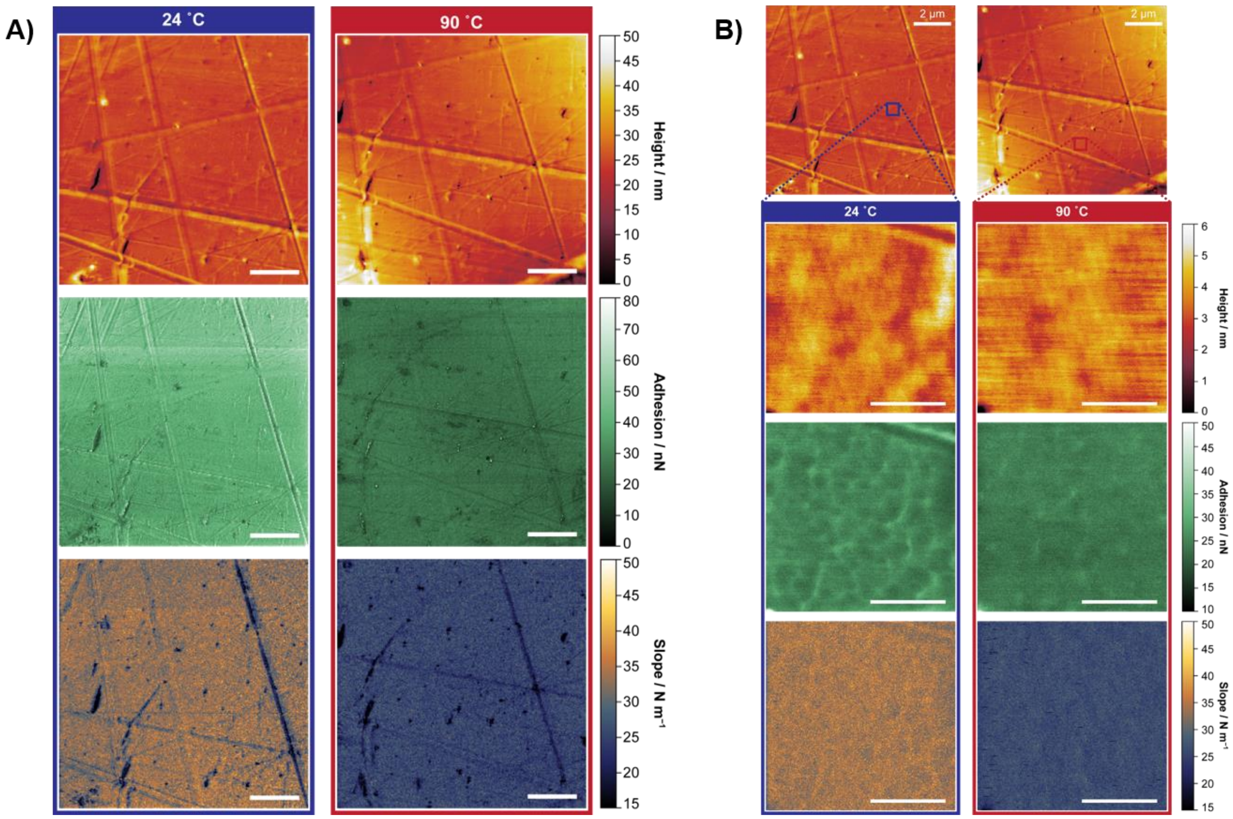

Supplementary Figure 39. C_3A-C_6T (82% *cis*) AFM analysis. A) Low resolution AFM images of the same area of a sample at temperatures below (24 °C, left hand column) and above (90 °C, right hand column) the T_g of the polymer. The height images (top row) show only small differences, probably due to slight heat deformation of the polymer. Increasing the temperature had only a minimal effect on the interaction of the AFM tip with the sample (adhesion, middle row), but a significant effect on the material's hardness (slope, bottom row): at higher temperatures, the material softened as expected. There were no visible changes in microstructure after heating, suggesting the absence of phase separation at this length scale. Scale bars = 2 μm. B) Higher resolution AFM images of the areas indicated in the 10 μm scans (A). As before, no significant morphological changes were observed with increasing temperature, but the material did become softer, as evidenced by the differences in the slope images (bottom row). Scale bars = 0.2 μm unless otherwise stated. Note that the colour scale used for the 10 μm height images is the same for both images. Also, the T_g decreases (*ca.* 70 – 80 °C) as the sample is aged under ambient (10 d for this sample) conditions and this is likely due to plasticization from water absorption. Refer to 1st run DSC data (Supplementary Figure 22 – Top). This is the reason the sample was heated to 90 °C and no significant differences were observed at 105 °C.

Supplementary Table 4. Physical, thermal and mechanical properties for polyamides with varied compositions.

Sample	[a]%*cis*	[b]M_w (kg mol^{-1})	$Đ_M$	[c]T_g (DSC)	T_d (5% loss)	[d]Modulus (MPa)	Yield Stress (MPa)	Strain at break (%)	Stress at break (MPa)
C_3A-C_{DEG}T$_{(cis)}$	78	140.4	3.84	64	334	1623.7 ± 77.0	78.1 ± 2.4	318 ± 70	58.8 ± 7.0
C_3A-C_{10}T$_{(cis)}$	78	80.5	2.77	71	334	927.7 ± 26.7	54.7 ± 1.1	313 ± 19	69.4 ± 3.7
C_3A-C_8T$_{(cis)}$	79	102.9	2.94	86	333	939.2 ± 67.0	59.2 ± 1.2	217 ± 11	63.7 ± 3.7
C_3A-C_6T$_{(cis)}$	82	104.6	3.35	98	334	1278.4 ± 42.1	70.4 ± 2.6	109 ± 28	65.9 ± 6.6
C_3A-C_6T$_{(73)}$	73	131.4	3.39	94	327	1219.3 ± 48.0	64.7 ± 1.5	158 ± 55	67.9 ± 4.2
C_3A-C_6T$_{(46)}$	46	111.8	3.84	83	328	1111.7 ± 19.6	60.9 ± 3.9	158 ± 45	58.1 ± 12.4
C_3A-C_6T$_{(35)}$	35	112.5	4.7	84	338	1052.5 ± 74.2	62.9 ± 5.1	138 ± 87	58.8 ± 16.3
C_3A-C_3T$_{(cis)}$	82	92.7	4.31	107	287	1547.8 ± 44.4	78.6 ± 1.1	24 ± 3	51.5 ± 2.7

[a]%*Cis* content determined by ^{1}H NMR anlysis. [b]M_w determined by SEC (DMF, 0.5 % w/w NH_4BF_4) analysis against poly(methyl methacrylate) (PMMA) standards. [c]T_g was determined from the second heating cycle. [d]Mechanical data were determined by tensile testing of independent samples (n ≥ 3).
